# Development and Implementation of In-House Pharmacogenomic Testing Program at a Major Academic Health System

**DOI:** 10.3389/fgene.2021.712602

**Published:** 2021-10-20

**Authors:** Pawel Mroz, Stephen Michel, Josiah D. Allen, Tim Meyer, Erin J. McGonagle, Rachel Carpentier, Alexandra Vecchia, Allyson Schlichte, Jeffrey R. Bishop, Henry M. Dunnenberger, Sophia Yohe, Bharat Thyagarajan, Pamala A. Jacobson, Steven G. Johnson

**Affiliations:** ^1^ Department of Laboratory Medicine and Pathology, University of Minnesota Medical School, Minneapolis, MN, United States; ^2^ Department of Experimental and Clinical Pharmacology, University of Minnesota College of Pharmacy, Minneapolis, MN, United States; ^3^ Institute for Health Informatics, University of Minnesota, Minneapolis, MN, United States; ^4^ Fairview Pharmacy Services, LLC, Minneapolis, MN, United States; ^5^ Department of Psychiatry, University of Minnesota Medical School, Minneapolis, MN, United States; ^6^ Mark R Neaman Center for Personalized Medicine Center, NorthShore University HealthSystem, Evanston, IL, United States

**Keywords:** pharmacogenomics, PGx, clinical decision support, personalized medicine, genetic variation, clinical implementation

## Abstract

Pharmacogenomics (PGx) studies how a person’s genes affect the response to medications and is quickly becoming a significant part of precision medicine. The clinical application of PGx principles has consistently been cited as a major opportunity for improving therapeutic outcomes. Several recent studies have demonstrated that most individuals (> 90%) harbor PGx variants that would be clinically actionable if prescribed a medication relevant to that gene. In multiple well-conducted studies, the results of PGx testing have been shown to guide therapy choice and dosing modifications which improve treatment efficacy and reduce the incidence of adverse drug reactions (ADRs). Although the value of PGx testing is evident, its successful implementation in a clinical setting presents a number of challenges to molecular diagnostic laboratories, healthcare systems, providers and patients. Different molecular methods can be applied to identify PGx variants and the design of the assay is therefore extremely important. Once the genotyping results are available the biggest technical challenge lies in turning this complex genetic information into phenotypes and actionable recommendations that a busy clinician can effectively utilize to provide better medical care, in a cost-effective, efficient and reliable manner. In this paper we describe a successful and highly collaborative implementation of the PGx testing program at the University of Minnesota and MHealth Fairview Molecular Diagnostic Laboratory and selected Pharmacies and Clinics. We offer detailed descriptions of the necessary components of the pharmacogenomic testing implementation, the development and technical validation of the in-house SNP based multiplex PCR based assay targeting 20 genes and 48 SNPs as well as a separate CYP2D6 copy number assay along with the process of PGx report design, results of the provider and pharmacists usability studies, and the development of the software tool for genotype-phenotype translation and gene-phenotype-drug CPIC-based recommendations. Finally, we outline the process of developing the clinical workflow that connects the providers with the PGx experts within the Molecular Diagnostic Laboratory and the Pharmacy.

## Introduction

According to the Center for disease Control (CDC) during the last decade almost half (45.8%) of the US population used one or more prescription drugs in the past 30 days (Martin et al., 2015). The Institute of Medicine estimates that there are ∼1.5 million preventable adverse drug reactions (ADRs) in the US annually, of which ∼100K result in death while accounting for an estimated $3.5 billion in additional health care costs(Sultana et al., 2013; Formica et al., 2018).

Pharmacogenomics (PGx) is a field of precision medicine that uses genetic variation to predict response to medications. Different responses and tolerability of individuals to the same drug at the same dose may occur as a result of interindividual differences in proteins involved in drug metabolism, transport or targets (Whirl-Carrillo et al., 2012). These differences may be inherited and occur mainly as a result of germline single nucleotide variants. There is growing evidence that most individuals (> 90%) harbor clinically actionable genetic variants that may affect how they respond to a medication affected by these variants(Ji et al., 2016). Among the 27 drugs frequently cited in ADRs ∼60% were associated with at least one drug-metabolizing enzyme with PGx variation (Alfirevic and Pirmohamed, 2017). In multiple well-conducted research studies (Pirmohamed, 2014), the results of PGx testing is important in guiding drug therapy choice and treatment dose efficacy and reduce the incidence of ADRs(Hoffman et al., 2014). Considering that most Americans will ultimately take medications throughout their lifetime and that 35% of Americans over 70 years of age use 5 or more prescription medications daily (Qato et al., 2016) routine implementation of PGx testing provides tremendous opportunities for improvement in clinical outcomes(Bell et al., 2014). The clinical implementation of PGx testing has been consistently cited as a major opportunity for improving patient care(Klein et al., 2017; Liu et al., 2020; Rigter et al., 2020). The NIH has funded numerous PGx initiatives such as the Pharmacogenomics Research Network (PGRN) (Shuldiner et al., 2013; Luzum et al., 2017; Barbarino et al., 2018) Implementing Genomics in Practice (IGNITE), (Weitzel et al., 2016; Cavallari et al., 2017; Levy et al., 2019), Clinical Pharmacogenetics Implementation Consortium (CPIC), (Relling and Klein 2011; Caudle et al., 2014), Pharmacogenomics Knowledge base (PharmGKB) (Barbarino et al., 2018; Klein and Ritchie 2018), Pharmacogene Variation Consortium (PharmVar) (Gaedigk et al., 2018) and other efforts to catalyze research and clinical implementation of PGx. While the scientific evidence is strong for PGx, implementation has been slow in healthcare organizations due to many factors including challenges for clinical diagnostics laboratories.

There is no single or dominant model for implementing PGx testing at a healthcare organization (Giri et al., 2019). A key decision for any healthcare organization seeking to implement a PGx program is whether to develop an in-house testing workflow or use the services of a reference laboratory. Many factors are important in making this crucial decision including resources, local expertise, equipment, expected clinical utilization of the assay, panel content, how the results are delivered into the EHR and the availability or potential to develop a clinical decision support (CDS) that is, relevant to the organization(Shuldiner et al., 2013). When deciding to develop an in-house PGx assay, the clinical molecular diagnostics laboratory faces an important decision about which technological platform to use, the platform’s flexibility when it comes to updating its assay composition and how it can be implemented in a cost effective manner. Different genotyping methods can be applied to analyze a patient’s PGx profile and the final design of the implemented PGx assay is extremely important. A well-designed PGx test must identify all significant polymorphisms that have an impact on the expression or function of drug-metabolizing enzymes, transporter proteins, and/or drug receptors. Therefore, the selection of the appropriate technology must be based on rigorous evaluation of several factors, including prior knowledge of the polymorphisms, sensitivity/specificity, sample requirements, and cost. Finally, the ability to provide data that can be directly integrated into the EHR either as discrete data or a PDF report or via access to the online portal outside the EHR needs to be considered. The implementation of the in-house assay provides the opportunity to develop CDS tools that can be customised to the local needs of patients and providers (Liu et al., 2021).

In this paper we describe a successful and highly collaborative implementation of the PGx testing program at the University of Minnesota and M Health Fairview and MHealth Fairview Molecular Diagnostic Laboratory (MDL) and selected Pharmacies and Clinics. We outline the development and technical validation of the in-house SNP targeting multiplex PCR based assay as well as a separate copy number assay for the CYP2D6 gene along with the process of PGx report design and the development of a software tool for genotype-phenotype translation and gene-phenotype-drug CPIC-based recommendations. Finally, we outline the process of developing the clinical workflow that connects the providers with the PGx experts within the molecular diagnostic laboratory and the pharmacy.

## Materials and Methods

### Multiplex PGx Assay

We developed a CPIC guided (cpicpgx.org, last access date 9/12/2021) multiplex PGx genotyping panel containing 48 TaqMan^®^ assays (Thermo Fisher Scientific, Waltham, MA, PN # 4351379) assessing 48 SNPs selected from 20 genes: ABCG2, CYP2B6, CYP2C19, CYP2C9, CYP2D6, CYP3A5, CYP4F2, DPYD, F2, F5, G6PD, GRIK4, HFE, MTHFR, SLC17A1, SLC22A12, SLCO1B1, TPMT, UGT1A1, VKORC1 (Supplementary Table S1 for details). All clinically implemented genes (CYP2B6, CYP2C19, CYP2C9, CYP2D6, CYP3A5, CYP4F2, DPYD, TPMT, UGT1A1, SLCO1B1) have a PharmGKB level of evidence 1A/1B with additional validated genes with evidence of 1A-2A/2B and 3 (F2). These SNPs were assayed on Fluidigm (South San Francisco, CA) 96.96 (BMK-M-96.96 GT) Dynamic Array Integrated Fluidics Chips (IFCs) used with a Fluidigm HX IFC controller, a Fluidigm FC1 cycler and a Fluidigm BioMark HD instrument.

### Positive Controls Design

To encompass each of the 3 possible allele combinations for all of the SNPs that were assayed, while also not occupying the majority of sample inlets of the IFC we constructed synthetic gBlocks^®^ (Integrated DNA Technologies, IDT, Coralville, Iowa). For each SNP tested, 2 × 251bp gBlocks were designed that centered on the SNP location itself. One version of the gBlock would contain the SNP base pair detected by a VIC TaqMan^®^ probe, while the other gBlock would contain the SNP base pair detected by a FAM TaqMan^®^ probe. To mitigate synthesis costs, these 251bp fragment designs were linked end-to-end with different 251bp fragments to become larger DNA fragments. The size of these fragments differed due to the number of fragments linked together and the SNP context sequences themselves. This resulted in 8 pools ranging in sizes 2510-2787bp assaying 10–12 different SNPs (See Supplementary Tables S2, S3).

All gBlocks detected by a VIC TaqMan probe were pooled together into one pool designated “VIC.” All gBlocks detected by FAM TaqMan probes were pooled together into one pool designated “FAM.” All “FAM” and “VIC” gBlocks were pooled together to obtain a heterozygous control designated “VICFAM.” All synthetic control pools were used at an input concentration of 1 million copies per microliter. All 8 synthetic control pools were utilized on the 96.96 IFCs for the PGx runs.

### Analytical Accuracy

The analytical accuracy of the multiplex PGx assay on the 96.96 Dynamic Array IFC was initially evaluated with 168 clinically unique DNA samples isolated from whole blood. These unique samples were previously or concurrently tested using validated orthogonal polymerase chain reaction-restriction fragment length polymorphism (PCR-RFLP, *n* = 88, 5 SNPs compared), or next generation sequencing (NGS, *n* = 34, 40 SNPs compared) methods (data not shown). Additionally, for interlaboratory and orthogonal method validation 46 DNA samples from outside institutions were used for comparison. Of the outside samples, a set of 10 samples was received from the Indiana University School of Medicine for blinded duplicate comparison of 31 SNPs. A set of four samples were received from Genomind, Inc. (King of Prussia, PA) for comparison of 2 rare CYP2C19 SNPs. Additionally, 24 samples from the Coriell Institute for Medical Research were used to evaluate accuracy (GeT-RM PGx, *n* = 21, 22 SNPs compared; 1000Genomes, *n* = 3, all 48 SNPs compared). Repository IDs of the Coriell samples from the GeT-RM Pharmacogenomics project are listed in Supplementary Table S5. A subset of 6 GeT-RM Coriell samples were also selected for orthogonal Sanger sequencing of 5 SNPs (data not shown). Finally, 8 in-house DNA samples that had previously been sent out for outside PGx testing (20 SNPs compared). Supplementary Table S6 highlights which SNPs were compared for each type of sample.

Calls for the PGx assay were categorized into True/False Positive/Negatives and No Calls. True positives (TP) were all concordant SNP genotyping calls that were heterozygous or homozygous for the minor or alternate allele in the SNP tested. True negatives (TN) were all concordant SNP genotyping calls that were homozygous for the major or functional allele in the SNP tested. False negatives (FN) were either homozygous calls for the major/functional allele when a heterozygous or homozygous call for the minor/altered allele was expected, or a heterozygous call when a homozygous call for the minor/altered allele was expected. False positives (FP) were either heterozygous or homozygous calls for the minor/altered allele when a homozygous call for the major/functional allele was expected, or a homozygous call for the minor/altered allele when a heterozygous call was expected. No Calls were instances where the sample failed to have a VIC and FAM relative fluorescence above 0.20 or were ambiguously located between the heterozygous cluster and a homozygous cluster of samples. All technical replicates of each unique sample were included in the tallying of true/false positives/negatives. Supplementary Table S7 shows the accuracy calls for each of the 168 samples on the Fluidigm platform.

### CNV Assay

A separately performed copy number variation (CNV) assay for CYP2D6 gene, run on a QuantStudio3 Real-Time PCR System (QS3) (Thermo Fisher, Waltham, MD) was also designed and validated. This CNV assay investigates gene expression levels in 3 different locations in CYP2D6 compared to expression levels in RNase P (see Supplementary Table S4), where CYP2D6 duplication or deletions are determined using the ΔΔCt method.

Three DNA samples obtained from the Coriell Institute for Medical Research with known CYP2D6 duplications, deletions or normal copy numbers were used as reference and standard samples. Sample #19 has a reported CYP2D6 genotype of *1/*1, with 2 copies of CYP2D6, and is used as the reference sample for the CNV run. Sample #144 has a CYP2D6 genotype of *4XN/*41, with 3 copies of CYP2D6, and is used as the duplication standard for this assay. Finally, sample #21 has a CYP2D6 genotype of *1/*5 (*5 is a whole gene deletion indicating only 1 copy of CYP2D6) and is used as the deletion standard for this assay. Sample #144 should have expression levels >1.3X compared to sample #19 for all 3 locations tested, and sample #21 should have expression levels <0.7X compared to sample #19 for all 3 locations tested for an assay run to be valid.

### Go4PGx Results Report Portal Development and Validation

We designed a web based portal, called Go4PGx, to provide CDS so that the results of the PGx test can be more easily interpreted and used by physicians and pharmacists. The CDS was provided in the form of a PDF report that details the genotype information, the gene phenotypes and gene-drug recommendations. The Go4PGx software was written in Ruby on Rails (https://rubyonrails.org/) and used a PostgreSQL database (https://www.postgresql.org/). The system supported uploading of variant SNP call data from a.csv file generated in the molecular diagnostics laboratory. The variant data was evaluated to produce diplotype matches based on information in the CPIC database [https://api.cpicpgx.org (V1), last date access 9/12/2021]. Go4PGx produced a web page and static portable document format (PDF) report that detailed genotype data and phenotype translation for all designated genes on the PGx testing panel, incorporation of a copy number variation (CNV) results for CYP2D6 as well as prescribing recommendations based on published CPIC guidelines.

We validated the Go4PGx algorithm by uploading 40 independent samples in duplicates with known genotype/diplotype. A report was generated for each of the samples and reviewed by the technician and a molecular genetic pathologist for haplotype/diplotype and phenotype calls for all the genes included on our assay and compared either to consensus calls or to manual calls made based on the CPIC allele tables.

### Go4PGx Report Usability Evaluation

In order to ensure that the PGx PDF Report would be easy to use, we conducted usability testing. An initial mockup of a PDF based report was created and included the following sections: “About this test” which offered introductory information about advantages and limitations of PGx testing; “Medication and Dosing Guidance for this Patient” which provided a graphical summary of findings; “Comprehensive Gene-Drug Interactions for this Patient” section which listed individual medications affected by the testing results together with resulted diplotype, CPIC based recommendation, level of CPIC evidence and a direct link to references; “Comprehensive Genotype-Phenotype Report for this Patient” section that listed gene, diplotype detected, phenotype, and CPIC based phenotype comment; “Go4PGx Pharmacogenomic Panel Gene and Variants” that provided the summary of all genes and SNPs tested for each gene including genomic position and nucleotide change as well as detected genotype/diplotype information; and Methodology, Limitations, and Comments sections.

Usability testing of the PGx PDF Report was subsequently performed. We recruited five volunteers to participate in 40-min virtual, Zoom-based usability testing sessions. The volunteers included a primary care physician, two clinical pharmacists, and two simulated patients. Participants were given a clinical scenario and a sample report populated with mock test results for a fictitious patient and three observers took detailed contemporaneous notes to describe each usability session. Feedback from the users was incorporated into the final design of the web portal and PGx PDF Report.

### Testing Workflow Development

**FIGURE 1:**
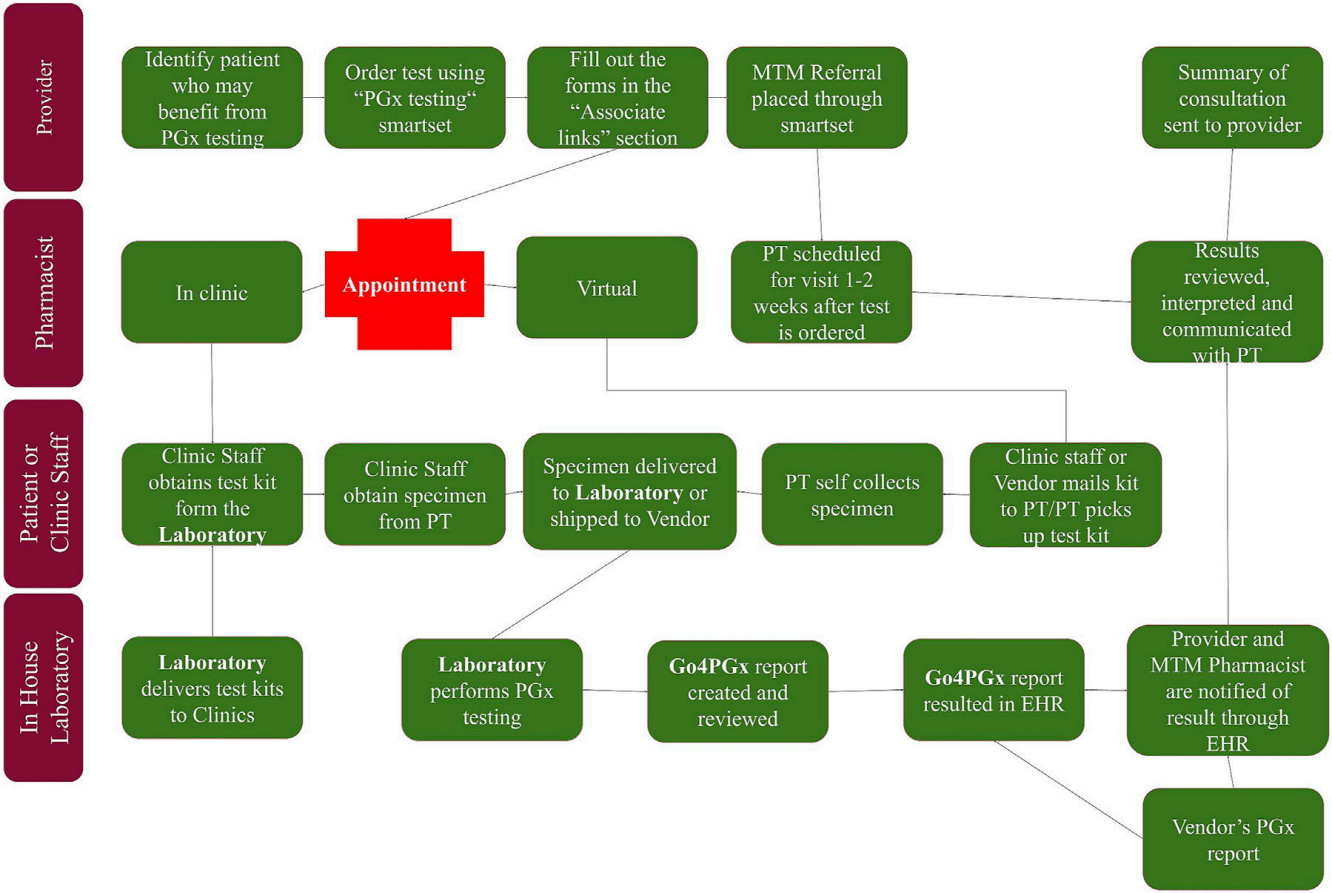
PGx testing workflow.

While in-house testing was being developed, workflow using external testing vendors was created to allow for pharmacogenomics testing to be utilized within the health system. Once in-house testing is implemented, this workflow allows for a smooth addition of the in-house testing option. The workflow began with providers identifying patients that may benefit from pharmacogenomic testing (see Figure 1). Testing guidelines were created to assist providers in identifying patients, but no strict inclusion or exclusion criteria were set. After discussing pharmacogenomic testing with the patient, the provider could place the order using an order set within the electronic health record. Once testing was completed, result notification was sent to a central laboratory team which uploaded the results into the electronic health record. After the results were available, a medication therapy management pharmacist met with each patient to review the results and make medication adjustments, if necessary.

### Education

Education to pharmacists consisted of in-depth information regarding good candidates for testing, pharmacogenetic testing interpretation and workflow overview. Education for clinic support staff focused on a high-level overview of the purpose of pharmacogenomic testing, the details of the workflow, and information about cost and billing. For providers, more detailed education was presented including information on clinical utility of pharmacogenomic testing along with guidance on which patients may benefit most from pharmacogenomic testing. A detailed description of the workflow including utilization of the order set was presented. Finally, providers were given a brief overview of interpreting pharmacogenomic test results. This education was presented either live during a virtual meeting or in a pre-recorded video. Additional workflow diagrams and tip sheets were sent to supplement the presentation. Pre and post surveys were completed to assess the provider education, and the pharmacist education was assessed using a post survey.

## Results

### Technical Validation of the Multiplex PCR PGx Assay

#### Analytical Accuracy

Out of the total of 11,788 calls that were compared for accuracy on this assay, there were 26 discrepancies: 18 false positives, and 8 false negatives (Table 1). All the false negatives and 15 of the 18 false positives could be explained by poor sample quality as those were old dried DNA samples needing to be reconstituted in water for repeat testing and showed poor global amplification (initial call rates <45.83%). Based on the validation results, poor quality samples that show poor amplification e.g., dried out samples are an inadequate sample type for this assay and such samples are not accepted for clinical use. Supplementary Figures 1–5 highlight the discrepant calls for each of the four poor quality samples.

**TABLE 1:** Accuracy calls total and for selected samples for the PGx panel on 96.96 IFC.

Sample number	TP	TN	FP	FN	No call
Total	2405	9176	18	8	181
18	20	198	5	4	13
23	29	208	2	0	1
37	5	71	4	2	38
40	25	85	4	2	4
151	48	83	0	0	1
154	48	101	1	0	0
163	32	104	2	0	6

Additionally, sample #151 was initially reported as having 6 false positives as well. In the original get-RM consortia project for which this validation is based, sample #151 was reported to have a consensus CYP2D6 genotype of *1/*10 (Pratt et al., 2010). Based on the SNPs tested in this assay, sample #151 should only be heterozygous positive for SNPs rs1135840 CYP2D6*2 (*39) and rs1065852 CYP2D6*10, which they were. However, sample #151 was also heterozygous positive for SNP rs16947 CYP2D6*2 (*34), which is not present in either *1 or *10 alleles. However, further studies characterizing sample #151 with Single Molecule Real Time (SMRT) sequencing also found the presence of this SNP, with uncertain phase in relation to the *10B allele (Qiao et al., 2016).

#### Specificity, Sensitivity, and Positive Predictive Value of Multiplex PGx Assay

Specificity was calculated as the ratio of true negatives over the sum of true negatives and false positives [TN/(TN + FP)]. Positive predictive value was calculated as the ratio of true positives over the sum of true and false positives [TP/(TP + FP)]. Finally, sensitivity was calculated as the ratio of true positives over the sum of true positives and false negatives [TP/(TP + FN)]. Table 2 illustrates the specificity, positive predictive value (PPV) and sensitivity for the PGx multiplex SNP panel on the 96.96 IFC.

**TABLE 2:** Specificity, positive predictive value, and sensitivity for PGx multiplex SNP panel on 96.96 IFC.

Specificity	PPV	Sensitivity
TN/(TN + FP)	TP/(TP + FP)	TP/(TP + FN)
99.80%	99.26%	99.67%

#### Precision

Precision of this assay on whole blood samples was calculated by comparing all calls in biologically identical samples run as technical replicates. These technical replicates were broken down into 3 main replicate types:

1. Technical replicates prepared by the same technologist on the same run (intra-run)
2. Technical replicates prepared by different technologists on different runs (inter-tech, 3 different Techs)
3. Technical replicates on different runs prepared either by the same technologist or different technologists (inter-run)

These replicate calls were measured against each other in a grand total per sample. Thus, the calls listed above are not exclusive to one group (i.e., if a sample was set up in duplicate on 2 separate runs, and had 3 concordant calls, but 1 discordant call, the discordant call would count as both an intra-run and inter-run discrepancy). Calls were not included in the precision data if they were No Calls.

171 clinically unique whole blood samples were compared across 13 runs on the 96.96 IFC for precision. Across those 13 runs, a total of 27,820 calls were compared. Of those 27,820 calls, there were 119 total discrepancies (Table 3). A similar approach was used to evaluate the precision of genotyping buccal swabs samples. Thirty five (35) unique samples of DNA, derived either from buccal swabs or blood samples of the same individuals were compared for precision for all SNPs on 4 separate runs (data not shown).

**TABLE 3:** Precision data between replicate samples.

Comparison type	Total calls	Concordant	Discordant	% Concordant
Total	27820	27701	119	99.57
Inter-run	15418	15303	115	99.25
Inter-tech	13270	13173	97	99.27
Intra-run	27820	27775	45	99.84

The discrepancies were categorized as 115 inter-run discrepancies, 97 inter-tech discrepancies, and 45 intra-run discrepancies. The discrepant base calls were analyzed in more detail and broken down into four types of causes: assay optimization, sample input quality, different extraction methods, and true discrepancy (see Table 4).

**TABLE 4:** Discrepancy breakdown.

Type	Discrepant	Assay optimization	Sample quality	Extraction method	True discrepancy
Total	119	38 (32%)	40 (34%)	40 (34%)	1 (< 1%)
Inter-run	115	36 (31%)	38 (33%)	40 (35%)	1 (1%)
Inter-tech	97	36 (37%)	20 (21%)	40 (41%)	1 (1%)
Intra-run	45	10 (22%)	28 (62%)	6 (13%)	1 (2%)

During initial runs of the validation, several assays exhibited poor relative VIC and FAM fluorescence with respect to the no target control, leading to indistinct allele clusters and ambiguous allele calls. Increasing the primer/probe concentration two-fold and thermal cycling the 96.96 IFC on an FC1 cycler mitigated the difficulties initially experienced.

As described in the accuracy section above, several samples showed poor global amplification (initial call rates <45.83%) that resulted in the discrepant calls. While those samples are included in the precisionanalsis based on the validation, such samples will not be accepted for resulting from this assay.

Samples included in the validation were derived from four different extraction methods. DNA was extracted from blood samples either with Qiagen DNeasy Blood and Tissue Kits, Promega Maxwell RSC Blood DNA Kits, or Promega Maxwell Whole Blood DNA Kits. Extracting blood with the Promega Maxwell Whole Blood kits proved problematic, as they had reduced yield when compared to the other methods of extraction, and therefore likely yielded less intact DNA. This likely led to an over approximation of how much DNA was in each sample, and therefore having reduced input into the 96.96 IFCs, so much so that the input of functional DNA was below the recommended input for the IFC.

**FIGURE 2:**
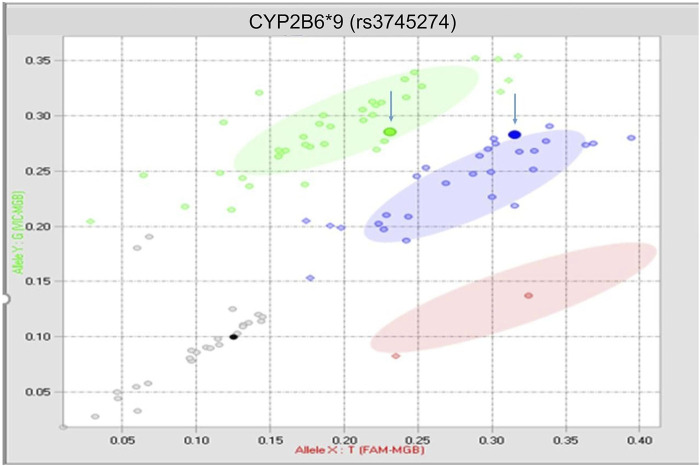
True discrepancy of 1 buccal swab sample. Both highlighted points should reside within the blue heterozygous cluster.

There was 1 discrepant call derived from a buccal swab sample (Figure 2) that occurred on the rs3745274 CYP2B6*9 assay after the assay had been optimized, and therefore could not be fully explained (inter-run 1%, *n* = 1; inter-tech 1%, *n* = 1; intra-run 2%, *n* = 1).

### Dynamic and Reference Range

The range of input DNA that produces reproducible test results was evaluated using a dilution series of samples with final DNA concentrations ranging from 2 to 200 ng/μL (input range amount of 5–500 ng).

Samples #31, 3100, and #107 were tested at concentrations of ∼2, 4, 20, 40, 100, and 200 ng/μL in triplicate. As seen in Table 5, the no call rates were non-zero with as little as 50 ng input (20 ng/μL). However, there was 1 intra-run discrepancy each for samples #31 and #107 at the 50 ng input, and an additional intra-run discrepancy for sample #31 at 100 ng input (40 ng/μL). Given the single discrepancy found at 40 ng/μL, the Fluidigm’s recommended concentration of 150 ng (60 ng/μL) was adopted as a minimum input into the IFC. To prevent disperse genotyping clusters from samples with far more available input, samples were arbitrarily assigned a maximum sample input concentration of 85 ng/μL (∼215 ng input). Any samples with a concentration >85 ng/μL were normalized down to 85 ng/μL before addition to assay reaction.

**TABLE 5:** The concordance percentages and no call rate.

Sample #	5 ng input		10 ng input		50 ng input		100 ng input		250 ng input		500 ng input	
	No call rate (%)	Concordance (%)	No call rate (%)	Concordance (%)	No call rate (%)	Concordance (%)	No call rate (%)	Concordance (%)	No call rate (%)	Concordance (%)	No call rate (%)	Concordance (%)
31	13.2	89.6	17.4	85.7	0.0	99.3	0.0	99.3	0.0	100.0	0.0	100.0
100	18.8	89.7	8.3	93.2	0.0	100.0	0.0	100.0	0.0	100.0	0.0	100.0
107	29.9	88.1	6.9	94.8	0.0	99.3	0.0	100.0	0.0	100.0	0.0	100.0

The reference values for positive and negative results for each SNP call were established in each SNP assay in each run based on the K-Means Clustering Method, where VIC and FAM intensity is normalized in relation to the No Target Control (NTC). Calls were made with a corresponding confidence interval (CI). Auto-calls are made for samples with ≥65% CI based on the K-means clustering. The low 65% confidence interval is used to flag poor globally amplifying samples for potential repeat. Samples are considered poor global amplifiers if their initial call rate is <45.83% (i.e., ≥4 auto calls as No Calls, percentage is based on 96 total assays, since 48 assays are empty on the IFC, the best initial call rate any sample can have is 50%). Furthermore, sample calls were deemed inadequate if the sample failed to have a VIC and FAM intensity of at least 0.20. These sample calls were changed to No Call, regardless of potential cluster pattern.

### Technical Validation of the CYP2D6 CNV Assay

#### Analytical Accuracy

The analytical accuracy of the CYP2D6 CNV assay on the QS3 was evaluated with 42 unique clinical samples across 11 runs. Calls for the CNV assay were categorized into True Positives, True Negatives, False Positives and False Negatives. True Positives (TP) were instances where <2 or >2 copies of CYP2D6 were called as expected. True Negatives (TN) were instances where 2 copies of CYP2D6 were called as expected. False Positives (FP) were instances where <2 or >2 copies of CYP2D6 were called, when 2 copies of CYP2D6 were expected. False Negatives (FN) were instances where 2 copies of CYP2D6 were called, when either <2 or >2 copies of CYP2D6 were expected. Copies numbers were inferred from the relative expression level for a given sample compared to reference sample 19 in the following ranges (<0.7 RE: 1 copy, 0.7–1.3 RE: 2 copies, 1.3–1.7 RE: 3 copies, 1.7–2.3 RE: four copies, >2.3 RE >4 copies).

CYP2D6 copy number calls were tabulated separately with respect to each region tested in CYP2D6, in order to further interrogate the presence of potential gene hybrids or rearrangements. Table 6 highlights the accuracy calls for each of the 42 samples on the Fluidigm platform.

**TABLE 6:** Accuracy calls for CYP2D6 CNV assay on QuantStudio3. Calls highlighted in yellow indicate initial discrepancies in copy number reported from the get-RM project versus that observed in this CNV assay.

Sample number	Reported CYP2D6 diplotype	CNV assay results						
		Exon 9 copies	Intron 6 copies	Intron 2 copies	TP	TN	FP	FN
1	*1/*1	2	2	2	0	3	0	0
2	*2A/*2A	2	2	2	0	3	0	0
3	*1/*3	2	2	2	0	3	0	0
19	*1/*2A	2	2	2	0	3	0	0
99	*1/*1	2	2	2	0	3	0	0
125	*1/*1	2	2	2	0	3	0	0
126	*2A/*4	2	2	2	0	3	0	0
127	*1/*4	2	2	2	0	3	0	0
143	*2XN/*17	3	3	3	3	0	0	0
144	*4XN/*41	3	3	3	33	0	0	0
145	*35/*41	2	2	2	0	3	0	0
146	*10/*10	2	4	4	0	1	2	0
147	*2/*10 (*2A/*36+*10B)	2	3	3	2	1	0	0
148	*1/*10	2	3	3	0	1	2	0
149	*2/*17	2	2	1	1	2	0	0
150	*1/*2	2	2	2	0	3	0	0
151	*1/*10 (*1A/*36+*10B)	2	3	3	2	1	0	0
152	*1/*2	2	2	2	0	3	0	0
153	*1/*4	2	2	2	0	3	0	0
154	*1/*41	2	2	2	0	3	0	0
155	*1XN/*2	3	3	3	3	0	0	0
156	*2/*2XN	3	3	3	3	0	0	0
157	DUP/*4/*2A	4	>4	4	6	0	0	0
158	*4/*35	2	2	2	0	3	0	0
159	*4/*5	1	1	1	3	0	0	0
160	*4/*41	2	3	3	0	1	2	0
161	*2/*3	2	2	2	0	3	0	0
162	*1/*1XN	3	3	3	3	0	0	0
163	*1/*6	2	2	2	0	3	0	0
164	*10/(*10[*36]) (*36+*10/*36+*10)	2	4	4	2	1	0	0
165	duplication	4	4	4	3	0	0	0
166	*17/*29	2	2	2	0	3	0	0
167	*1/*1	2	2	2	0	33	0	0
168	*1/*5	1	1	1	33	0	0	0
169	*1/*35	2	2	2	0	3	0	0
170	*1/*9	2	2	2	0	3	0	0
171	*1/*41	2	2	2	0	3	0	0
172	*1/*5	1	1	1	3	0	0	0
173	*2/*5	1	1	1	9	0	0	0
174	*5/*9	1	1	1	3	0	0	0
175	*1/*1	2	2	2	0	3	0	0
176	*1/*41	2	2	2	0	3	0	0
—				Total: 225	112	107	6	0

**FIGURE 3:**
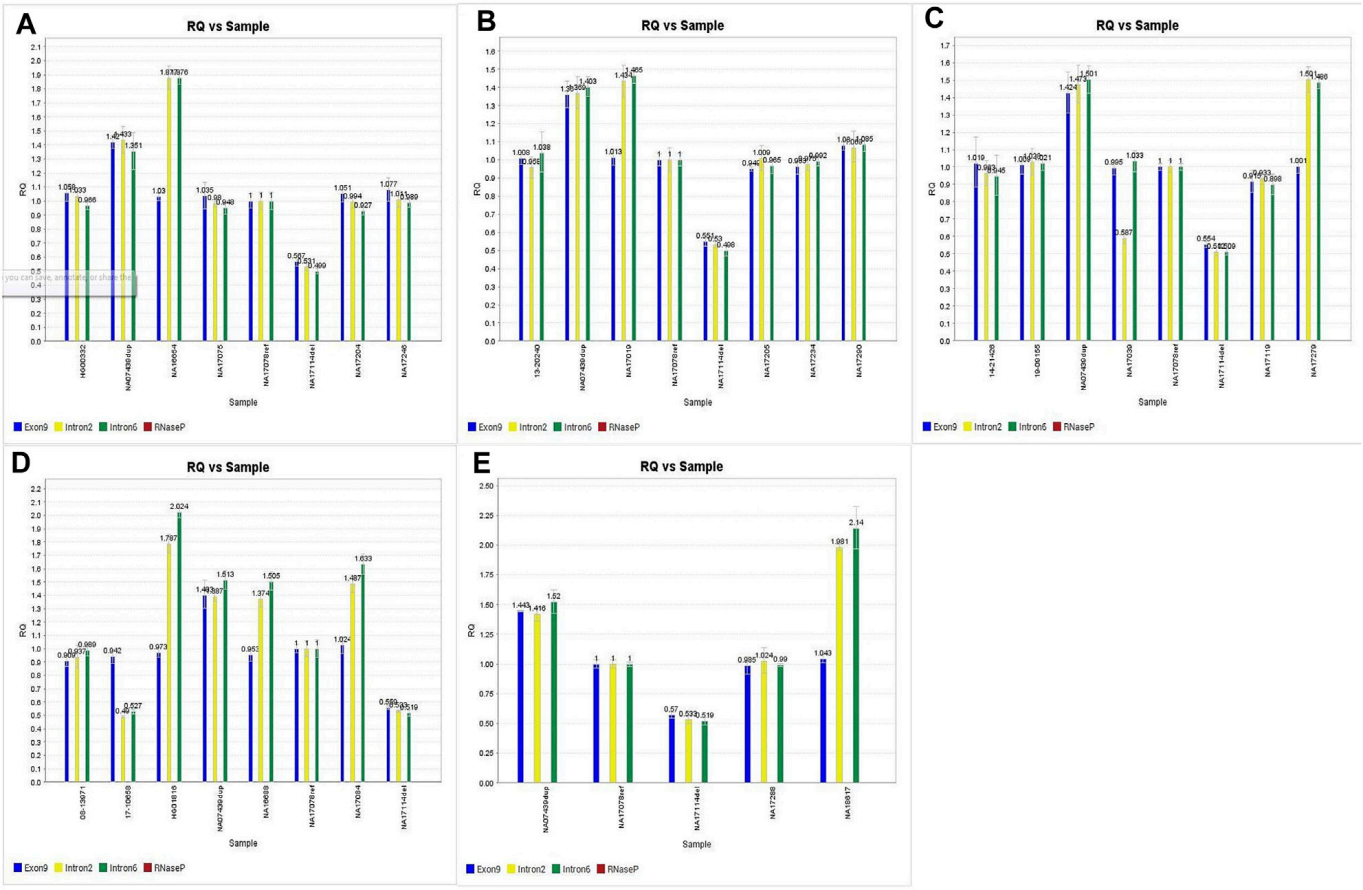
A,B,C. Discrepant CNV calls for samples 146, 148 and 160. D.E. Imbalanced CNV calls for samples 147, 20 and 164 subsequently confirmed through orthogonal studies.

Out of the 225 calls that were compared for accuracy on this assay, there were 6 false positives from 3 samples (#146, #148, and #160). In all 3 cases, samples had concordant copy numbers based on relative Exon 9 expression, but elevated CYP2D6 copies based on the relative expression in both Intron 2 and Intron 6 (See Figures 3A–C).

Coriell samples #147, #20, and #164 also exhibited this same expression pattern (See Figures 3D,E). These samples have been further characterized with single molecule real time (SMRT) long read sequencing, confirming that the disparities between the Exon 9 and Introns 2/6 copy numbers are the result of gene rearrangements (Qiao et al., 2016; Gaedigk et al., 2019). Additionally, the drop in Intron 2 relative expression in sample #149 has been demonstrated in subsequent studies (Kolb et al., n.d), likely the cause of allelic dropout. Finally, while the difference in degree of elevated relative expression of Intron 6 in sample #157 has no bearing on the accuracy of the call; there was reproducibly elevated Intron 6 relative expression compared to Exon 9 and Intron 2 throughout this validation.

Given the not insignificant percentage of the population harboring CYP2D6 structural variants (∼13%) (Del Tredici et al., 2018) [3], and the other Coriell samples with the elevated Intron 2/6 expression pattern having their copy numbers confirmed through subsequent study, it is likely all 6 false positives are in fact true positives, and samples #146, #148 and #160 also harbor structural rearrangements of CYP2D6. However, further characterization of Coriell samples #146, #148, and #160 have not been performed since the GeT-RM project to confirm this assertion.

The assay specificity, sensitivity and positive predictive value (as defined in the TaqMan^®^ SNP Genotyping section above) for the CNV assay are summarized in Table 7.

**TABLE 7:** Specificity, positive predictive value, and sensitivity for CNV assay on QS3.

Specificity	PPV	Sensitivity
TN/(TN + FP)	TP/(TP + FP)	TP/(TP + FN)
94.69	94.92	100.00

### Precision

Five unique samples of DNA were compared across 11 runs to determine precision of the CYP2D6 CNV assay on the QS3. Intra-run, inter-run and inter-tech replicates (*n* = 2) were used as defined in the previous section. A total of 114 inter-run calls were made, with all calls being concordant for a concordance of 100%. Furthermore, a subset of 33 of those 100% concordant calls were considered inter-tech replicates, while a further subset of 6 of those 100% concordant calls were considered intra-run replicates (Table 8).

**TABLE 8:** Precision data for CYP2D6 CNV assay.

Comparison type	Total calls	Concordant	Discrepant	% Concordant
Inter-run	114	114	0	100.00
Inter-tech	33	33	0	100.00
Intra-run	6	6	0	100.00

A dynamic range was not investigated for the CYP2D6 CNV assay, as samples are normalized down to 5 ng/μL, and a total of 20 ng input was used in the CNV assay reaction.

### Go4PGx PDF Report Usability Study

**FIGURE 4:**
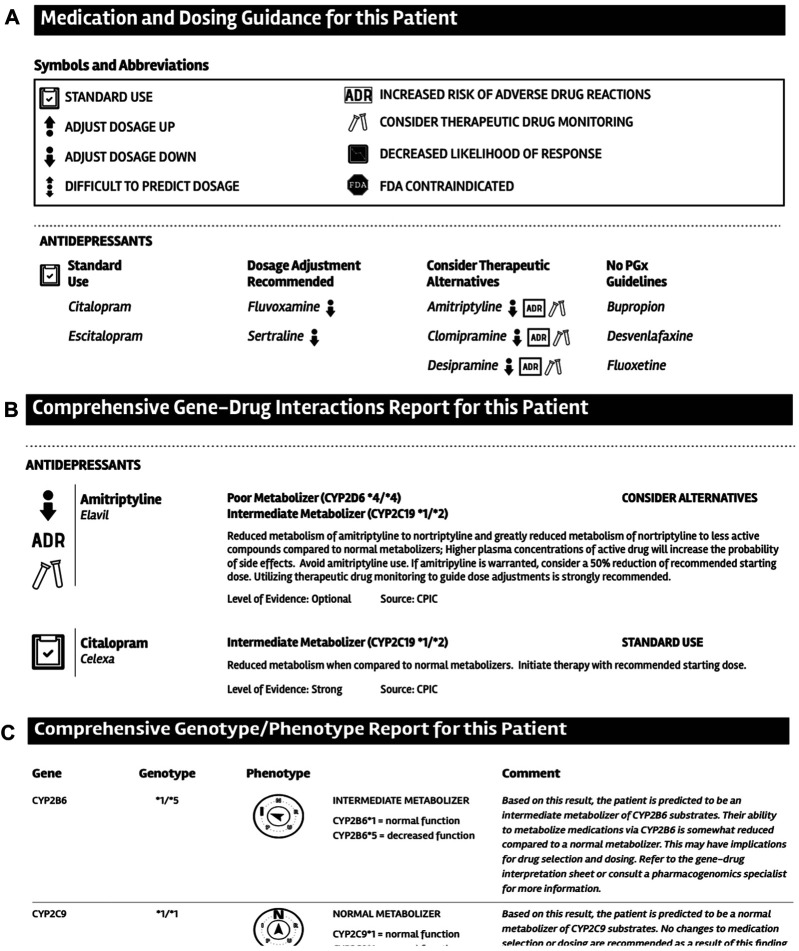
Examples of selected sections design from the PGx report. A. Medication and dosing guidance for this patient section. B. Comprehensive Gene-Drug Interactions Report for this Patient section. C. Comprehensive Genotype-Phenotype Report for this Patient section.

We performed the usability testing based on standard procedures from the University of Minnesota Usability Lab. In our usability test we collected feedback from 5 users. Each user was presented with a mockup PGx PDF eport (see Figure 4) and asked to review the report and highlight any possible problems or suggestions for improvement they identified. The majority of problems encountered by the users were subjective in nature and included confusion with the language of text or headers as well as some of the icons used. Users highlighted issues with information being perceived to be mis-ordered, or information that was not included in the report. Specific examples included suggestions to change the order that the results were being displayed so that first the genotype information summary, then the gene-drug recommendations and finally the more detailed genotype information is displayed. Additional suggestions included simplification of graphics and icons to be more understandable. Finally, users suggested that the wording to patients on the first page of the report should be simplified and pharmacogenomic terms and jargon removed to make it more easily comprehended by patients. This feedback from the usability testing was subsequently used to change a number of aspects of the final report design.

### Education

In order to implement PGx testing workflow in primary care clinics within the health system, education was presented to pharmacists, providers and clinic support staff at four pilot sites. A total of 16 providers attended live training during a virtual meeting, and 23 additional providers received pre-recorded videos of the training. Pre- and post-surveys were completed to assess the effects of education sessions. 12 providers responded to the pre-survey and nine providers responded to the post-survey. The providers’ experience with pharmacogenomic testing prior to education was very minimal, with eight of the 12 providers having never ordered a pharmacogenomic test previously. Providers were asked to rate their confidence in their ability to identify patients that may benefit from pharmacogenomics testing on a scale of one to five (1 being not at all confident and 5 being extremely confident). The median response increased from 2.5 to 4 after receiving education. When asked if any part of the education was unclear or confusing, all of the providers responded “no.”

A total of 14 medication therapy management pharmacists completed training. Nine out of 14 pharmacists completed the post survey (64%). Seven of the 9 responders indicated they would be comfortable if they were to get a PGx referral that day (78%). The two pharmacists who indicated they were not comfortable described that they would simply need to review the training materials again prior to seeing a patient for a PGx consult. All of the responders indicated that they knew where to find information to answer clinical questions about PGx. Eight of the 9 responders indicated they knew where to find answers to workflow related questions (89%).

### Go4PGx Report and Portal Validation

During the Go4PGx report and portal validation we tested 40 independent samples. Each sample included genotyping results for 9 genes, 32 SNPs, and a CNV information for the CYP2D6 gene. Each sample was uploaded to the portal in duplicate and each duplicate was analyzed independently and subsequently compared. There were no discrepancies in haplotype/diplotype/phenotype calls between the sample duplicates and all results were concordant and called correctly by the portal algorithm. In one case (#151, see above) the portal did not generate the result for the CYP2D6 gene (which was the expected behavior) and the result was manually reviewed and reported as indeterminate. The manual interpretation as “indeterminate” triggers the following CPIC database based phenotype comment that appears on the report: “the expected phenotype for this individual cannot be determined based on the CYP2D6 result. While no changes to medication selection or dosing are recommended as a result of this finding for CYP2D6, the individual should be monitored closely for medication response. Consult the laboratory or a pharmacogenomics specialist for more information.” It is estimated that individuals carrying CYP2D6 haplotypes with an unknown, uncertain, or uncurated function (in our report referred to as indeterminate) range from 2 to 9%, with a study of the UK Biobank finding that 3.4% of individuals carry haplotypes that cannot be mapped to a predefined function (McInnes et al., 2020). Therefore, for these patients, pharmacogenomic-guided therapy for drugs metabolized by CYP2D6 (e.g., CPIC and DPWG dosing guidelines) cannot be used and such recommendation is reflected in our report.

## Discussion

There have been a number of medical centers in the United States and beyond that have undertaken the difficult effort of implementing pharmacogenomic testing in recent years, (Gottesman et al., 2013; Bielinski et al., 2014; Shuldiner et al., 2014; Arwood et al., 2016; Eadon et al., 2016; Rosenman et al., 2017; van der Wouden et al., 2017; Cavallari et al., 2018; Rigter et al., 2020), including several medical centers in the state of Minnesota (Bishop et al., 2021). The successful implementation of the PGx testing program remains, however, a challenging enterprise. It requires numerous available resources, coordination of multiple complex steps, communication with various stakeholders and a group of visionary individuals that will fuel the implementation process with their enthusiasm. In this paper we describe a highly collaborative process of development and validation of a CPIC guided PGx testing built on the joint efforts of stakeholders from University of Minnesota College of Pharmacy, Institute for Health Informatics and Department of Laboratory Medicine and Pathology as well as M Health Fairview Molecular Diagnostic Laboratory and Clinical Pharmacies. The final products of this collaboration are a multiplex SNP and CYP2D6 CNV assays that are validated for use on both whole blood or buccal swabs samples. Additionally, we describe a process of design and validation of a Go4PGx reporting portal and a PDF based PGx report. Finally, we describe the process of pharmacy workflow implementation for in-house and send-out PGx testing as well as the education efforts targeting local providers.

**FIGURE 5:**
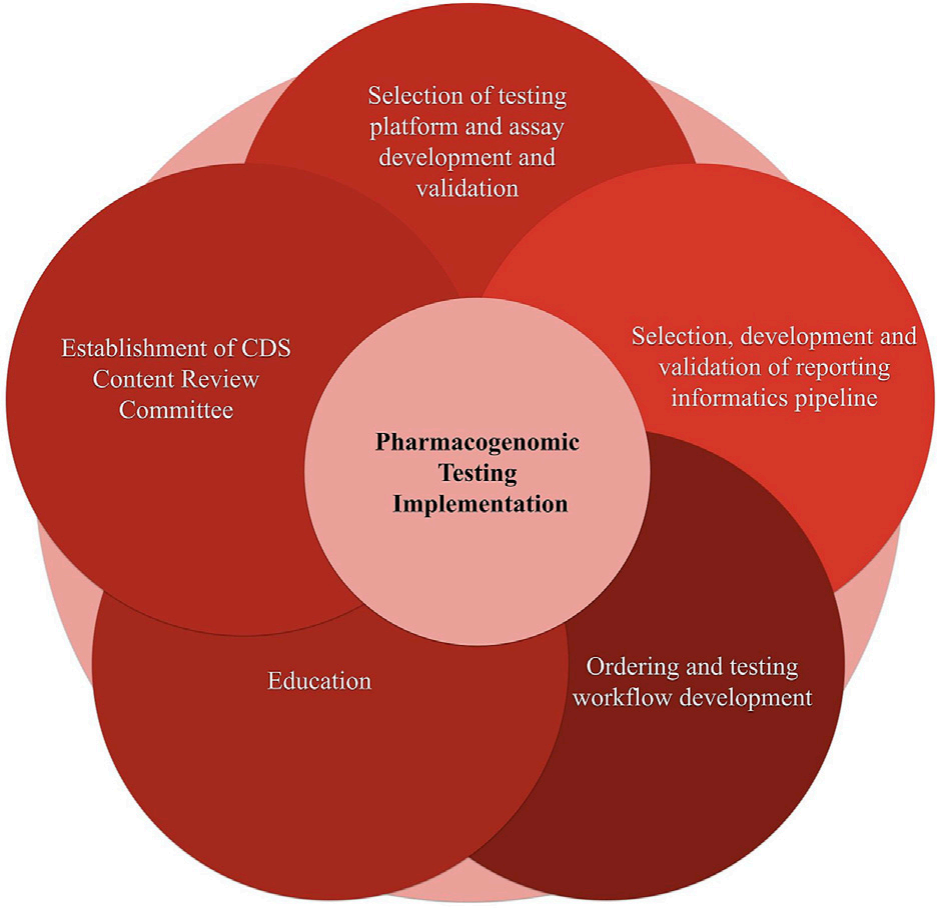
Components of the pharmacogenomic testing implementation.

We divided the process of pharmacogenomic implementation into the following independent, yet interconnected steps (see Figure 5). Selection of testing platform and assay development and validation; 2. Selection and development of a reporting informatics pipeline; 3. Ordering and testing workflow development; 4. Education; 5.Establishment of Clinical Decision Support Content Review Committee (CCRC). Below we discuss the implementation considerations of each of the aforementioned steps.

### Selection of Testing Platform and Assay Development and Validation

Several technological options exist and have been described for clinical grade PGx genotyping including real-time polymerase chain reaction (RT-PCR), restriction length fragment polymorphism (RFLP) analysis, microarray, PCR followed by Sanger sequencing, and genome, exome or gene panel library preparation followed by Next Generation Sequencing (NGS)—also known as “massively-parallel sequencing.” Each genotyping platform has non-overlapping strengths and weaknesses as well as comes with unique advantages and disadvantages [see detailed review here (Hippman and Nislow, 2019)]. After reviewing several commercially available options including two microarray assays (Infinium Global Diversity Array with Enhanced PGx-8 v1.0 by Illumina and PharmacoScan Assay by Applied Biosystems) as well as FDA-cleared genotyping platforms [select examples reviewed here (Arwood et al., 2016)] we elected to validate a multiplex real-time polymerase chain reaction based laboratory developed assay. This decision was mostly based on cost effectiveness of the RT-PCR genotyping platform as well as prior development experience of a similar research grade assay (Wen et al., 2020; Sun et al., 2021). The decision on gene and allele selection that makes up the composition of the initial assay was one of the most difficult tasks in the PGx testing implementation process. The initial composition of the SNP based multiplex assay derives from our recent research publications (Wen et al., 2020; Sun et al., 2021) where we initially multiplexed 20 genes and 48 SNPs to test the most common and PGx relevant star alleles and variants for these genes. However, aware of the significant discrepancies between clinically and commercially offered gene panels (Bousman and Dunlop 2018) as well as our limited local clinical PGx testing expertise, for the first iteration of the clinical grade PGx genotyping panel we tailored the assay down to 9 genes, 32 SNPs, and 32 star alleles (see Supplementary Table S1 for details). Our decision was in part dictated by the series of Association for Molecular Pathology (AMP) published recommendations that define the minimum set (tier 1) and expanded set (tier 2) of alleles that should be included on a pharmacogenomic assay. Recommendations have been published for CYP2C19 (Pratt et al., 2018), CYP2C9 (Pratt et al., 2019), as well as for warfarin genotyping (Pratt et al., 2020), with recommendations for additional genes expected to be published in the coming years. For our initial clinical rollout, we included AMP tier 1 variants for genes with published recommendations along with a subset of tier 2 SNPs for selected genes. For genes without published AMP recommendations we relied on the CPIC level A evidence as well as PharmGKB level 1A evidence. We also limited the initial assay based on the availability of the selected SNPs and phenotype recommendations in the CPIC guidelines database. Additionally, the assay cannot describe with any confidence any CYP2D7 conversions or CYP2D6/CYP2D7 structural variations and the CYP2D6 CNV assay cannot determine the specific increased number of gene copies beyond duplication. Despite those limitations we are able to deliver CPIC based gene-phenotype-drug recommendations on 33 medications from 14 therapeutic classes to help patients cared for by at least seven clinical specialties (see Supplementary Table S8 for details), making our assay comparable to other medical centers and commercial vendors offerings (Caudle et al., 2018; Haga and Kantor 2018; Tilleman et al., 2019; Bishop et al., 2021). As we build on our PGx testing expertise the next iteration of the assay will be planned and the increase in both gene and allele counts, with consideration for other CPIC recommended genes, genes that occur with reasonably high frequency in the US population, those that are associated with a phenotype that can influence medication selection (e.g., Factor 2 and Factor 5) and variants that may be more common in some of the unique ancestral populations (e.g., East African, Hmong) that currently reside in the Minneapolis-St. Paul metro area, will be considered.

### Selection and Development of a Reporting Informatics Pipeline

Another major task, outside of the assay development and validation, was to decide how to effectively report the genotyping results and PGx recommendations to the providers within the healthcare system environment to ensure the results can be easily used. This is particularly challenging as PGx evidence is rapidly evolving and literature is being generated daily with varying levels of clinical strength. Therefore, the next key requirement for clinical implementation of the PGx testing program was to decide on an appropriate CDS system (Cohen and Felix, 2014). Recently, leading medical centers have begun offering CDS services allowing clinical laboratories to generate in-house laboratory reports with the help of the external CDS support (Cannon and Allen, 2000; Garg et al., 2005). We began the process by investigating the selected available commercial offerings by inviting vendors (Translational Software, Coriell Life Sciences, and YouScript) that have developed robust CDS tools to give software demonstrations and explain the software onboarding process. Each company offered different levels of CDS ranging from static PDF reports through a stand-alone portal to a full CDS EHR integration with different levels of clinical, research and pricing options. After considering several commercial vendors’ offerings however, we decided on developing an in-house reporting infrastructure called Go4PGx that will initially allow us to generate a passive CDS in the form of a static PDF report with an immediate second-phase goal to develop an active CDS (Devine et al., 2014) that will be available within the clinical workflow of medication ordering. This decision was partly based on the recently released CPIC database, the availability of local expertise in building CDS software through collaboration with the University of Minnesota Institute for Health Informatics as well as the realization that by building local reporting infrastructure we accumulate the necessary know-how that will be invaluable in future expansion of the PGx testing program.

We therefore designed a web-based portal that allows reporting on PGx variants of interest from a genetic data set generated by the molecular diagnostic laboratory and evaluating diplotype matches based on CPIC guidelines using the CPIC published database [https://api.cpicpgx.org (V1), last date access 9/12/2021]. The initial design required defining the panel in the administrative interface consisting of the Genes, Haplotypes, and SNPs that would match the capabilities of the PGx genotyping assay. The Go4PGx portal takes the panel information and builds all the possible combinations of SNP/allele results and subsequently evaluates each haplotype from the CPIC rules database. Each combination is given a unique signature to match results against. When a new result file is uploaded from the molecular laboratory, Go4PGx evaluates all the possible phased combinations of SNP/allele combinations based on the genotyping results, and generates a signature to compare against all possible signatures to determine the list of candidate haplotypes and diplotypes. Once a detailed diplotype match review is performed, a PDF report is generated which contains the phenotype and medication guidance information consistent with the CPIC database at the time of the report generation (last accessed 8/23/2021). Each report is automatically dated and versioned with that information. The resulting report can be subsequently downloaded from the portal and uploaded into the EHR by the laboratory.

We used usability testing to define key features in the report design. The usability testing, although performed only on a selected group of potential users, allowed for improvement in several sections of the final PDF report design. For instance, we simplified the layout of the report and removed several icons including the entire section called “Medication and Dosing Guidance for this Patient” that included several icons that were thought to be too confusing. Since our main workflow for the result includes MTM pharmacists based on the received feedback, we reorganized the order of the sections so that the “Comprehensive Genotype/Phenotype Report” section is first followed by the “Comprehensive gene-drug interactions” section. Finally, we worked with the MHealth Fairview Patient Education and Technology Office to improve the patient centered language of the “About this test” section of the report that provides a brief overview of the PGx testing.

### Ordering and Testing Workflow Development

In collaboration with pharmacy services, we developed an ordering and testing workflow. The initial workflow was developed for the purpose of enabling send out testing to commercial laboratories and subsequently replicated once the in-house testing capabilities were achieved. The workflow begins with providers including medical doctors, nurse practitioners, and physician assistants identifying patients that may benefit from pharmacogenomic testing (see Figure 1). After discussing pharmacogenomic testing with the patient, the provider can place the order using an order smartset within the electronic health record that lists both send out and in-house testing options. Once the molecular diagnostic laboratory or vendor receives the order and the sample, the genotyping is performed. The MDL laboratory performs the genotyping on the described assay. For in-house testing, the laboratory workflow includes the uploading of the genotyping results in the form of a.csv file to the Go4PGx portal followed by initial review of the results inside the portal by a trained technologist who also runs the genotyping assay. The portal allows for manual review of genotypes, interpreted diplotypes, and phenotypes that are computed by the software as well as the final report. It also allows the technologist to flag any results that may need additional review. If there are no issues with the reports, the technologist will finalize the report and upload the final report to the EHR that will be reviewed and signed out by the molecular genetic pathologist. If there are issues, the technologist will flag the report and alert the molecular genetic pathologist. If the portal is not able to compute the diplotype based on the provided genotype it will provide all the possible genotype options and there is an option for the pathologist to manually assign the final diplotype including an option to call a diplotype indeterminate which triggers appropriate CPIC based phenotype comment.

Once testing is completed and the results are available, a medication therapy management pharmacist meets with each patient to review the results and make medication adjustments, if necessary. These medication adjustments may be made by the pharmacist using a broad collaborative practice agreement (CPA) between the medication therapy management pharmacists and primary care providers. This CPA was already in existence within the health system prior to the implementation of this pharmacogenomics testing workflow. The pharmacist sends a summary note of their clinical assessment and any medication changes to the ordering provider. The complete PGx report is available in the electronic medical record for the provider to review if they desire, although they are not required to do so.

### Education

Providers’ views of PGx testing can impact clinical implementation. Most providers are interested in genetic testing (Hauser et al., 2018); however, the clinical acceptance of PGx testing, while increasing, is still relatively limited. This may be in part due to frustrations with the complex nomenclature and limited predictive nature of the testing, or perhaps due to inconsistencies in published data. For widespread clinical acceptance, a clear and understandable testing workflow must be developed so all providers are comfortable ordering and integrating PGx results into their practices. In order to aid with this process we enlisted local expertise in the form of well-trained pharmacists who designed the ordering-testing-reporting-clinical correlation pipeline as well as developed ordering and training materials and led the training sessions with providers. The education efforts were initially targeted towards selected providers and clinics that were early adopters of the send out PGx testing and subsequently expanded on a system-wide scale. Our education efforts were focused on the very practical aspects of the PGx testing. These were slide based presentation (Microsoft PowerPoint) sessions and included the following domains: background on PGx (including advantages and limitations of testing), how to identify patients that may benefit from testing, how to explain PGx to a patient, overview of the workflow and available testing options and broad overview of result interpretation. The education sessions allowed for identification of several initial barriers to the system-wide roll out of PGx testing. These included difficulty with ensuring that providers completed the hour-long training and maintaining provider and staff knowledge given the current infrequency of PGx ordering.

### Clinical Decision Support Content Review Committee

The development and implementation of the pharmacogenomic testing program the MHealth Fairview was part of an even larger project entitled, “Toward Pharmacogenomics-Enabled Healthcare at Statewide Scale: Implementing Precision Medicine” through a University of Minnesota Grand Challenge Research investment. As part of this larger project a multi-institutional Go4PGx CDS Content Review Committee (CCRC) was established. The CCRC was modeled after a hospital P and T committee and consists of representatives from clinical pharmacy, molecular genetic pathology, primary care, ambulatory, and acute care physicians, genetic counseling, and healthcare IT. The CCRC provides an interdisciplinary governance and oversight process allowing for the ongoing maintenance of Go4PGx CDS content. The CCRC oversight and expertise allows for a scientific and structured review and potential modification of the existing CDS content beyond current CPIC database content, particularly if newer scientific evidence becomes available in the literature or in instances where additional language from FDA labeling may be desired. It also allows for the review of previously generated reports on a biannual basis or every time CPIC releases a critical database update.

## Summary and Conclusion

In summary, we describe here our experience of development of in-house PGx testing program and the minimum necessary steps for its successful implementation. In the process we had to face and overcome several systemic barriers that included initial lack of the local laboratory expertise in the PGx testing, lack of the available IT infrastructure to provide CDS for pharmacogenomics, initial institutional hesitancy to commit resources for development and implementation of PGx testing and uncertainties regarding the ability to get reimbursed for the PGx testing. We relied however, on a highly collaborative approach that allowed us to build a multi-institutional team that leveraged a variety of expertise to overcome those barriers and minimise any potential shortcomings. The College of Pharmacy provided PGx expertise and developed the CCRC governance process, the MHealth Fairview Pharmacies developed testing and clinical workflows as well as provider education materials, the Institute of Health Informatics created the Go4PGx portal, and the Molecular Diagnostics Laboratory developed and validated PGx assay and Go4PGx reports. All efforts were coordinated through the members of the Department of Laboratory Medicine and Pathology. Having support of all administrative levels including health-care system administration, pharmacy, and laboratory medicine including commitment to finance parts of the ongoing development helped establish the legitimacy of the implementation efforts and allowed for this broad collaborative effort to be successful.

As the knowledge of PGx relevant genetic variants and clinical applicability constantly expands and as testing becomes more inexpensive, health care systems will face not only the need for developing similar programs but also seek to continuously update their PGx testing panels to keep up with the pace of scientific discoveries or to customize the assay to fit local providers and patients needs. In our process we relied on a highly collaborative approach to take the full advantage of the unique expertise of all local stakeholders. We believe that such a collaborative understanding of the necessity of developing local knowhow and PGx expertise was the key to the successful implementation and maintenance of such a complex clinical testing program.

## Data Availability

The original contributions presented in the study are included in the article/Supplementary Material, further inquiries can be directed to the corresponding authors.
