# Supplementary material for: Development and Implementation of In-House Pharmacogenomic Testing Program at a Major Academic Health System
Source: Front Genet. 2021 Oct 20;12:712602. doi: 10.3389/fgene.2021.712602 (PMC8564018; doi:10.3389/fgene.2021.712602)
Supplement: Supplementary file 1 [file DataSheet1.docx]

**Supplemental Materials:**

**Supplemental Table 1 . PGx TaqMan SNP Genotyping Assay Overview**

| **Assay Name** | **Gene** | **GRCh37 Coordinates** | **Alteration** | **ThermoFisher Assay ID** | **VIC Labelled Allele** | **FAM Labelled Allele** | **dbSNP Minor/Alternate Allele Frequency*** |
| --- | --- | --- | --- | --- | --- | --- | --- |
| rs2231142 ABCG2 | ABCG2 | 4:89052323 | G>T | C__15854163_70 | G | **T** | T=0.12465 |
| rs3745274 CYP2B6*9 | CYP2B6 | 19:41512841 | G>T | C___7817765_60 | G | **T** | T=0.28312 |
| rs4244285 CYP2C19*2 | CYP2C19 | 10:96541616 | G>A | C__25986767_70 | **A** | G | A=0.17640 |
| rs4986893 CYP2C19*3 | CYP2C19 | 10:96540410 | G>A | C__27861809_10 | **A** | G | A=0.00538 |
| rs28399504 CYP2C19*4 | CYP2C19 | 10:96522463 | A>G | C__30634136_10 | A | **G** | G=0.00200 |
| rs56337013 CYP2C19*5 | CYP2C19 | 10:96612495 | C>T | C__27861810_10 | C | **T** | T=0.00001 |
| rs72552267 CYP2C19*6 | CYP2C19 | 10:96535210 | G>A | C__27531918_10 | **A** | G | A=0.00033 |
| rs41291556 CYP2C19*8 | CYP2C19 | 10:96535173 | T>C | C__30634130_30 | **C** | T | C=0.00155 |
| rs12248560 CYP2C19*17 | CYP2C19 | 10:96521657 | C>T | C____469857_10 | C | **T** | T=0.19899 |
| rs1799853 CYP2C9*2 | CYP2C9 | 10:96702047 | C>T | C__25625805_10 | C | **T** | T=0.09202 |
| rs1057910 CYP2C9*3 | CYP2C9 | 10:96741053 | A>C | C__27104892_10 | **C** | A | C=0.04520 |
| rs28371686 CYP2C9*5 | CYP2C9 | 10:96741058 | C>G | C__27859817_40 | C | **G** | G=0.00330 |
| rs9332131 CYP2C9*6 | CYP2C9 | 10:96709040 | delA | C__32287221_20 | **del** | A | delA=0.00076 |
| rs7900194 CYP2C9*8 | CYP2C9 | 10:96702066 | G>A | C__25625804_10 | **A** | G | A=0.01736 |
| rs28371685 CYP2C9*11 | CYP2C9 | 10:96740981 | C>T | C__30634132_70 | **T** | C | T=0.00359 |
| rs16947 CYP2D6*2 (*34) | CYP2D6 | 22:42523943 | G>A | C__27102425_10 | **A** | G | A=0.37647 |
| rs1135840 CYP2D6*2 (*39) | CYP2D6 | 22:42522613 | C>G | C__27102414_10 | C | **G** | G=0.54901** |
| rs35742686 CYP2D6*3 | CYP2D6 | 22:42524244 | delT | C__32407232_50 | T | **del** | delT=0.01221 |
| rs3892097 CYP2D6*4 | CYP2D6 | 22:42524947 | C>T | C__27102431_D0 | C | **T** | T=0.13858 |
| rs5030655 CYP2D6*6 | CYP2D6 | 22:42525086 | delA | C__32407243_20 | A | **del** | delA=0.00792 |
| rs1065852 CYP2D6*10 | CYP2D6 | 22:42526694 | G>A | C__11484460_40 | **A** | G | A=0.20875 |
| rs28371706 CYP2D6*17 | CYP2D6 | 22:42525772 | G>A | C___2222771_A0 | G | **A** | A=0.0585 |
| rs28371725 CYP2D6*41 | CYP2D6 | 22:42523805 | C>T | C__34816116_20 | C | **T** | T=0.08073 |
| rs776746 CYP3A5*3 | CYP3A5 | 7:99270539 | T>C | C__26201809_30 | T | **C** | C=0.71078** |
| rs2108622 CYP4F2*3 | CYP4F2 | 19:15990431 | C>T | C__16179493_40 | C | **T** | T=0.27314 |
| rs67376798 DPYD D949V | DPYD | 1:97547947 | T>A | C__27530948_10 | **A** | T | A=0.00285 |
| rs3918290 DPYD*2A | DPYD | 1:97915614 | C>T | C__30633851_20 | C | **T** | T=0.00280 |
| rs55886062 DPYD*13 | DPYD | 1:97981343 | A>C | C__11985548_10 | A | **C** | C=0.00032 |
| rs1799963 Factor 2 | F2 | 11:46761055 | G>A | C___8726802_20 | **A** | G | A=0.00995 |
| rs6025 Factor 5 | F5 | 1:169519049 | C>T | C__11975250_10 | C | **T** | T=0.01959 |
| rs1050828 G6PD*Asahi | G6PD | X:153764217 | C>T | C___2228686_20 | C | **T** | T=0.00906 |
| rs72554665 G6PD*Bangkok Noi | G6PD | X:153760484 | C>A | ANGZGGG | C | **A** | A=0.00045 |
| rs5030868 G6PD*Mediterranean | G6PD | X:153762634 | G>A | C___2228708_20 | **A** | G | A=0.00255 |
| rs1954787 GRIK4 c.83-10039T>C | GRIK4 | 11:120663363 | T>C | C___2138789_20 | **C** | T | C=0.46328 |
| rs1799945 HFE H63D | HFE | 6:26091179 | C>G | C___1085600_10 | C | **G** | G=0.10924 |
| rs1800562 HFE C282Y | HFE | 6:26093141 | G>A | C___1085595_10 | G | **A** | A=0.03321 |
| rs1801133 MTHFR | MTHFR | 1:11856378 | G>A | C___1202883_20 | G | **A** | A=0.31486 |
| rs1183201 SLC17A1 | SLC17A1 | 6:25823444 | A>T | C___1911034_10 | A | **T** | T=0.66453** |
| rs505802 SLC22A12 | SLC22A12 | 11:64357072 | T>C | C____925802_10 | **C** | T | C=0.46461 |
| rs4149056 SLCO1B1*5 | SLCO1B1 | 12:21331549 | T>C | C__30633906_10 | **C** | T | C=0.13288 |
| rs1800462 TPMT*2 | TPMT | 6:18143955 | C>G | C__12091552_30 | C | **G** | G=0.00174 |
| rs1800460 TPMT*3B | TPMT | 6:18139228 | C>T | C__30634116_20 | **T** | C | T=0.02804 |
| rs1142345 TPMT*3C | TPMT | 6:18130918 | T>C | C_____19567_20 | **C** | T | C=0.03673 |
| rs1800584 TPMT*4 | TPMT | 6:18131012 | C>T | C__12091550_20 | C | **T** | T=0.00003 |
| rs887829 UGT1A1*80 | UGT1A1 | 2:234668570 | C>T | C___2669357_10 | C | **T** | T=0.34885 |
| rs9923231 VKORC1*2 -c.1639 G>A | VKORC1 | 16:31107689 | C>T | C__30403261_20 | C | **T** | T=0.33619 |
| rs9934438 VKORC1*2 6484G>T | VKORC1 | 16:31104878 | G>A | C__30204875_10 | **A** | G | A=0.33635 |
| rs7294 VKORC1*3 | VKORC1 | 16:31102321 | C>T | C___7473918_10 | C | **T** | T=0.3828 |

Highlighted alleles (bold) are the minor or alternate allele. Underlined genes indicate genes on the initially rolled out clinical assay.

*dsSNP minor allele frequencies determined from dbSNP database with the most numerous cases (<https://www.ncbi.nlm.nih.gov/snp/>)

**Frequencies listed that are greater than 50% are by definition not the minor allele frequencies, but instead the allele frequencies with altered functions or the alternate allele listed in dbSNP for the gene containing the SNP.

**Supplemental Table 2. gBlocks for each synthetic control in Version 1. Alterations are the highlighted base. VIC probes will bind to bases highlighted in green. FAM probes will bind to bases highlighted in red. Rows highlighted in yellow indicate 2 tested SNPs being within 251bp of one another.**

| **NCBI SNP Reference** | **VIC SNP Sequence** | **FAM SNP Sequence** | **Genomic Location** | **“Stitched” Fragment** |
| --- | --- | --- | --- | --- |
| **rs1801133 MTHFR** | GGCCCCTCACCTGGATGGGAAAGATCCCGGGGACGATGGGGCAAGTGATGCCCATGTCGGTGCATGCCTTCACAAAGCGGAAGAATGTGTCAGCCTCAAAGAAAAGCTGCGTGATGATGAAATCG**G**CTCCCGCAGACACCTTCTCCTTCAAGTGCTTCAGGTCAGCCTCAAAGCTCCCTGCTTCGGGGTGGCCTTTGGGGTAACCTGCCAATAGGGATGACAGTCAGGAGAGGCTGGCCTCCACCTGTTCA | GGCCCCTCACCTGGATGGGAAAGATCCCGGGGACGATGGGGCAAGTGATGCCCATGTCGGTGCATGCCTTCACAAAGCGGAAGAATGTGTCAGCCTCAAAGAAAAGCTGCGTGATGATGAAATCG**A**CTCCCGCAGACACCTTCTCCTTCAAGTGCTTCAGGTCAGCCTCAAAGCTCCCTGCTTCGGGGTGGCCTTTGGGGTAACCTGCCAATAGGGATGACAGTCAGGAGAGGCTGGCCTCCACCTGTTCA | chr1:11856378 (GRCh37.p13) | A |
| **rs6025 Factor 5** | TGACAATTACTGTTCTCTTGAAGGAAATGCCCCATTATTTAGCCAGGAGACCTAACATGTTCTAGCCAGAAGAAATTCTCAGAATTTCTGAAAGGTTACTTCAAGGACAAAATACCTGTATTCCT**C**GCCTGTCCAGGGATCTGCTCTTACAGATTAGAAGTAGTCCTATTAGCCCAGAGGCGATGTCTCTCATGATGTCCACGTCACTGTAGTATGGTCTTGTTAAGCACTGGGCATCATTTTCTGTGGGT | TGACAATTACTGTTCTCTTGAAGGAAATGCCCCATTATTTAGCCAGGAGACCTAACATGTTCTAGCCAGAAGAAATTCTCAGAATTTCTGAAAGGTTACTTCAAGGACAAAATACCTGTATTCCT**T**GCCTGTCCAGGGATCTGCTCTTACAGATTAGAAGTAGTCCTATTAGCCCAGAGGCGATGTCTCTCATGATGTCCACGTCACTGTAGTATGGTCTTGTTAAGCACTGGGCATCATTTTCTGTGGGT | chr1:169519049 (GRCh37.p13) | A |
| **rs67376798DPYD D949V** | CTTTCTGCCGTAAAAACAAGAAGAAACATGTCTCATAGCATTCTAATTCCAGCAGGATTCTTACCTGGTAGCCAGAATCATTACAGGTCATGTAGCATTTACCACAGTTGATACACATTTCTTCA**A**CAATCATAGCCACAACTTGCTCTACGTTGCTCAATTCACCAAATGTTCCAAGGTACTGCAGTGCTTTTCCTATTACATCCTAAAAATAGCCACTGAATTACTTAGCAAGCTCATTTTAAAACATT | CTTTCTGCCGTAAAAACAAGAAGAAACATGTCTCATAGCATTCTAATTCCAGCAGGATTCTTACCTGGTAGCCAGAATCATTACAGGTCATGTAGCATTTACCACAGTTGATACACATTTCTTCA**T**CAATCATAGCCACAACTTGCTCTACGTTGCTCAATTCACCAAATGTTCCAAGGTACTGCAGTGCTTTTCCTATTACATCCTAAAAATAGCCACTGAATTACTTAGCAAGCTCATTTTAAAACATT | chr1:97547947 (GRCh37.p13) | A |
| **rs3918290 DPYD*2A** | AAGCAACTGGCAGATTCTTTAATAAAATATACACATTAATATTTATAAGCCTATGAATTGGATGTTTAAATAAACATTCACCAACTTATGCCAATTCTCTTGTTTTAGATGTTAAATCACACTTA**C**GTTGTCTGGAAAGTCAGCCTTTAGTTCAGTGACACTTTGACACCAATATGCAGCCGTTTTCTCACTGATGAGCTCAATATTCAGAAAGGAGCTTTGTCCAGGGCCATACATGGGGCCAGAGGTGG | AAGCAACTGGCAGATTCTTTAATAAAATATACACATTAATATTTATAAGCCTATGAATTGGATGTTTAAATAAACATTCACCAACTTATGCCAATTCTCTTGTTTTAGATGTTAAATCACACTTA**T**GTTGTCTGGAAAGTCAGCCTTTAGTTCAGTGACACTTTGACACCAATATGCAGCCGTTTTCTCACTGATGAGCTCAATATTCAGAAAGGAGCTTTGTCCAGGGCCATACATGGGGCCAGAGGTGG | chr1:97915614 (GRCh37.p13) | A |
| **rs55886062 DPYD*13** | TGTTTATACCTTAATTAAAATATATGATAGACATTTCTATATGACTTCAATAATATTTTCTTACCTTATCAAGAGAGAAAGTTTTGGTGAGGGCAAAACCCCATCCAGCTTCAAAAGCTCTTCGA**A**TCATTGATGTGCTGGTGGCTGGAGTTGCGCTAGCAAGACCAAAAGGATTTATAAACTTCAATCCGGCCATTTCTACACTAATGTCCACCAGATCAATAGGAGTGTAAAAGAGGGGTAGTTCAGGC | TGTTTATACCTTAATTAAAATATATGATAGACATTTCTATATGACTTCAATAATATTTTCTTACCTTATCAAGAGAGAAAGTTTTGGTGAGGGCAAAACCCCATCCAGCTTCAAAAGCTCTTCGA**C**TCATTGATGTGCTGGTGGCTGGAGTTGCGCTAGCAAGACCAAAAGGATTTATAAACTTCAATCCGGCCATTTCTACACTAATGTCCACCAGATCAATAGGAGTGTAAAAGAGGGGTAGTTCAGGC | chr1:97981343 (GRCh37.p13) | A |
| **rs4149056 SLCO1B1*5** | AATAATTAAGAGTTTACAAGTAGTTAAATTTGTAATAGAAATGCTAAAATTAATGTTTAAAATGAAACACTCTCTTATCTACATAGGTTGTTTAAAGGAATCTGGGTCATACATGTGGATATATG**C**GTTCATGGGTAATATGCTTCGTGGAATAGGGGAGACTCCCATAGTACCATTGGGGCTTTCTTACATTGATGATTTCGCTAAAGAAGGACATTCTTCTTTGTATTTAGGTAATGTACACAAAATAT | AATAATTAAGAGTTTACAAGTAGTTAAATTTGTAATAGAAATGCTAAAATTAATGTTTAAAATGAAACACTCTCTTATCTACATAGGTTGTTTAAAGGAATCTGGGTCATACATGTGGATATATG**T**GTTCATGGGTAATATGCTTCGTGGAATAGGGGAGACTCCCATAGTACCATTGGGGCTTTCTTACATTGATGATTTCGCTAAAGAAGGACATTCTTCTTTGTATTTAGGTAATGTACACAAAATAT | chr12:21331549 (GRCh37.p13) | A |
| **rs7294 VKORC1*3** | TTGCTCAGAACCTTCCCTCCCTGGGCAATGGAAAGAGCTTTGGAGACCAGCCCATGGGGACAGAGTCAGAGGCACTGGGTGTAAAAAAGAGCGAGCGTGTGGCACATTTGGTCCATTGTCATGTG**C**GGGTATGGCAGGAGGAGGGGGTAATCTAGAAGCCCCACATCTAGGGCCTTCTAGGGACCCAGATATGCCCCCTTAGGCAAGGCTCACATGCCAAAGCAAAGCAGATGAGGTCAGCCTGGCTTGGG | TTGCTCAGAACCTTCCCTCCCTGGGCAATGGAAAGAGCTTTGGAGACCAGCCCATGGGGACAGAGTCAGAGGCACTGGGTGTAAAAAAGAGCGAGCGTGTGGCACATTTGGTCCATTGTCATGTG**T**GGGTATGGCAGGAGGAGGGGGTAATCTAGAAGCCCCACATCTAGGGCCTTCTAGGGACCCAGATATGCCCCCTTAGGCAAGGCTCACATGCCAAAGCAAAGCAGATGAGGTCAGCCTGGCTTGGG | chr16:31102321 (GRCh37.p13) | A |
| **rs9934438 VKORC1*2 6484G>T** | GGTGGGGTGGGGTGGAACCAGGTTAGGACTGTCAACCCAGTGCCTTGGACCCTGCCCGAGAAAGGTGATTTCCAAGAAGCCACCTGGGCTATCCTCTGTTCCCCGACCTCCCATCCTAGTCCAAG**A**GTCGATGATCTCCTGGCACCGGGCACCTTTGGCCACGTCAGGATTCCATGTCACTGACCCTATCCTCCCCTCTCCCCAGACCAGGCCCGGACGTGGCTACTCCGTAGGCCCTGCTTTTCATCTTA | GGTGGGGTGGGGTGGAACCAGGTTAGGACTGTCAACCCAGTGCCTTGGACCCTGCCCGAGAAAGGTGATTTCCAAGAAGCCACCTGGGCTATCCTCTGTTCCCCGACCTCCCATCCTAGTCCAAG**G**GTCGATGATCTCCTGGCACCGGGCACCTTTGGCCACGTCAGGATTCCATGTCACTGACCCTATCCTCCCCTCTCCCCAGACCAGGCCCGGACGTGGCTACTCCGTAGGCCCTGCTTTTCATCTTA | chr16:31104878 (GRCh37.p13) | A |
| **rs9923231 VKORC1*2 -c.1639 G>A** | TTGTATTTTTAGTAGAGACAGGGTTTCACCATGTTGGCCAGGCTTGTCTTAAACTCCTGACCTCAAGTGATCCACCCACCTCGGCCTCCCAAAATGCTAGGATTATAGGCGTGAGCCACCGCACC**C**GGCCAATGGTTGTTTTTCAGGTCTTCTCTTGCTTGACTTCCCAGAGGGATCCCTTACTGTTGCACCTACCCTTCTGGGAACTCTCTTCCTCTGGCGTCTGTGATATTTCCCTCTCCTGCTGGCTC | TTGTATTTTTAGTAGAGACAGGGTTTCACCATGTTGGCCAGGCTTGTCTTAAACTCCTGACCTCAAGTGATCCACCCACCTCGGCCTCCCAAAATGCTAGGATTATAGGCGTGAGCCACCGCACC**T**GGCCAATGGTTGTTTTTCAGGTCTTCTCTTGCTTGACTTCCCAGAGGGATCCCTTACTGTTGCACCTACCCTTCTGGGAACTCTCTTCCTCTGGCGTCTGTGATATTTCCCTCTCCTGCTGGCTC | chr16:31107689 (GRCh37.p13) | A |
| **rs887829 UGT1A1*80** | AGTGAGCAGGCAGTACCGGGGGAGCTGTGGAGTGGGCACTCTTACAGGTTTCCATGGCGAAAGCGGGGGTACAGTTGTGTTCTTTTCTTTCTAAAAGGCTTTCTAAAAAGCCTTCTGTTTAATTT**C**TGGAAAAGAAGCCTAACTTGTTCACTACATAGTCGTCCTTCTTCCTCTCTGGTAACACTTGTTGGTCTGTGGAAATACTAATTTAATGGATCCTGAGGTTCTGGAAGTACTTTGCTGTGTTCACT | AGTGAGCAGGCAGTACCGGGGGAGCTGTGGAGTGGGCACTCTTACAGGTTTCCATGGCGAAAGCGGGGGTACAGTTGTGTTCTTTTCTTTCTAAAAGGCTTTCTAAAAAGCCTTCTGTTTAATTT**T**TGGAAAAGAAGCCTAACTTGTTCACTACATAGTCGTCCTTCTTCCTCTCTGGTAACACTTGTTGGTCTGTGGAAATACTAATTTAATGGATCCTGAGGTTCTGGAAGTACTTTGCTGTGTTCACT | chr2:234668570 (GRCh37.p13) | A |
| **rs2108622 CYP4F2*3** | AGTTGTGTGTGTCTTTGAGGGAGGTGATGTTGGATACTCCTGATCAAAACCCTGCCCCCTCCTCTAGGAGCCTTGGAATGGACAAAAACAGAGAGAGGGGCCCCGCACCTCAGGGTCCGGCCACA**C**AGCTGGGTTGTGATGGGTTCCGAAAACACTGATGAGGCAGATAATGCCTGTGGGAGAGAAGGGAGCAGTCAGGAGAAGGCCTCCTTCACTGAGGGGCCCCTCTTCCTACCCAGGAGACTCCTCCC | AGTTGTGTGTGTCTTTGAGGGAGGTGATGTTGGATACTCCTGATCAAAACCCTGCCCCCTCCTCTAGGAGCCTTGGAATGGACAAAAACAGAGAGAGGGGCCCCGCACCTCAGGGTCCGGCCACA**T**AGCTGGGTTGTGATGGGTTCCGAAAACACTGATGAGGCAGATAATGCCTGTGGGAGAGAAGGGAGCAGTCAGGAGAAGGCCTCCTTCACTGAGGGGCCCCTCTTCCTACCCAGGAGACTCCTCCC | chr19:15990431 (GRCh37.p13) | B |
| **rs3745274 CYP2B6*9** | CTGAGTGATGGCAGACAATCACACAGAGATAGGTGACAGCCTGATGTTCCCCAGGCACTTCAGTCTGTGTCCTTGACCTGCTGCTTCTTCCTAGGGGCCCTCATGGACCCCACCTTCCTCTTCCA**G**TCCATTACCGCCAACATCATCTGCTCCATCGTCTTTGGAAAACGATTCCACTACCAAGATCAAGAGTTCCTGAAGATGCTGAACTTGTTCTACCAGACTTTTTCACTCATCAGCTCTGTATTCGG | CTGAGTGATGGCAGACAATCACACAGAGATAGGTGACAGCCTGATGTTCCCCAGGCACTTCAGTCTGTGTCCTTGACCTGCTGCTTCTTCCTAGGGGCCCTCATGGACCCCACCTTCCTCTTCCA**T**TCCATTACCGCCAACATCATCTGCTCCATCGTCTTTGGAAAACGATTCCACTACCAAGATCAAGAGTTCCTGAAGATGCTGAACTTGTTCTACCAGACTTTTTCACTCATCAGCTCTGTATTCGG | chr19:41512841 (GRCh37.p13) | B |
| **rs28399504 CYP2C19*4** | ATAGTGGGCCTAGGTGATTGGCCACTTTATCCATCAAAGAGGCACACACACTTAATTAGCATGGAGTGTTATAAAAAGCTTGGAGTGCAAGCTCACGGTTGTCTTAACAAGAGGAGAAGGCTTCA**A**TGGATCCTTTTGTGGTCCTTGTGCTCTGTCTCTCATGTTTGCTTCTCCTTTCAATCTGGAGACAGAGCTCTGGGAGAGGAAAACTCCCTCCTGGCCCTACTCCTCTCCCAGTGATTGGAAATATC | ATAGTGGGCCTAGGTGATTGGCCACTTTATCCATCAAAGAGGCACACACACTTAATTAGCATGGAGTGTTATAAAAAGCTTGGAGTGCAAGCTCACGGTTGTCTTAACAAGAGGAGAAGGCTTCA**G**TGGATCCTTTTGTGGTCCTTGTGCTCTGTCTCTCATGTTTGCTTCTCCTTTCAATCTGGAGACAGAGCTCTGGGAGAGGAAAACTCCCTCCTGGCCCTACTCCTCTCCCAGTGATTGGAAATATC | chr10:96522463 (GRCh37.p13) | B |
| **rs41291556/rs72552267 CYP2C19*8/CYP2C19*6** | TTCTCGGGCAGAGCTTGGCCCATCCACATGGCTGCCCAGTGTCAGCTTCCTCTTTCTTGCCTGGGATCTCCCTCCTAGTTTCGTTTCTCTTCCTGTTAGGAATCGTTTTCAGCAATGGAAAGAGA**C**GGAAGGAGATCCGGCGTTTCTCCCTCATGACGCTGC**A**GAATTTTGGGATGGGGAAGAGGAGCATTGAGGACCGTGTTCAAGAGGAAGCCCGCTGCCTTGTGGAGGAGTTGAGAAAAACCAAGGGTGGGTGAACATACTCTCTATCACTGACCTTTCTGGACT | TTCTCGGGCAGAGCTTGGCCCATCCACATGGCTGCCCAGTGTCAGCTTCCTCTTTCTTGCCTGGGATCTCCCTCCTAGTTTCGTTTCTCTTCCTGTTAGGAATCGTTTTCAGCAATGGAAAGAGA**T**GGAAGGAGATCCGGCGTTTCTCCCTCATGACGCTGC**G**GAATTTTGGGATGGGGAAGAGGAGCATTGAGGACCGTGTTCAAGAGGAAGCCCGCTGCCTTGTGGAGGAGTTGAGAAAAACCAAGGGTGGGTGAACATACTCTCTATCACTGACCTTTCTGGACT | chr10:96535173/ chr10:96535210 (GRCh37.p13) | B |
| **rs4986893 CYP2C19*3** | GGCTGTGCTCCCTGCAATGTGATCTGCTCCATTATTTTCCAGAAACGTTTCGATTATAAAGATCAGCAATTTCTTAACTTGATGGAAAAATTGAATGAAAACATCAGGATTGTAAGCACCCCCTG**A**ATCCAGGTAAGGCCAAGTTTTTTGCTTCCTGAGAAACCACTTACAGTCTTTTTTTCTGGGAAATCCAAAATTCTATATTGACCAAGCCCTGAAGTACATTTTTGAATACTACAGTCTTGCCTAGA | GGCTGTGCTCCCTGCAATGTGATCTGCTCCATTATTTTCCAGAAACGTTTCGATTATAAAGATCAGCAATTTCTTAACTTGATGGAAAAATTGAATGAAAACATCAGGATTGTAAGCACCCCCTG**G**ATCCAGGTAAGGCCAAGTTTTTTGCTTCCTGAGAAACCACTTACAGTCTTTTTTTCTGGGAAATCCAAAATTCTATATTGACCAAGCCCTGAAGTACATTTTTGAATACTACAGTCTTGCCTAGA | chr10:96540410 (GRCh37.p13) | B |
| **rs4244285 CYP2C19*2** | AGTTTTAAATTACAACCAGAGCTTGGCATATTGTATCTATACCTTTATTAAATGCTTTTAATTTAATAAATTATTGTTTTCTCTTAGATATGCAATAATTTTCCCACTATCATTGATTATTTCCC**A**GGAACCCATAACAAATTACTTAAAAACCTTGCTTTTATGGAAAGTGATATTTTGGAGAAAGTAAAAGAACACCAAGAATCGATGGACATCAACAACCCTCGGGACTTTATTGATTGCTTCCTGAT | AGTTTTAAATTACAACCAGAGCTTGGCATATTGTATCTATACCTTTATTAAATGCTTTTAATTTAATAAATTATTGTTTTCTCTTAGATATGCAATAATTTTCCCACTATCATTGATTATTTCCC**G**GGAACCCATAACAAATTACTTAAAAACCTTGCTTTTATGGAAAGTGATATTTTGGAGAAAGTAAAAGAACACCAAGAATCGATGGACATCAACAACCCTCGGGACTTTATTGATTGCTTCCTGAT | chr10:96541616 (GRCh37.p13) | B |
| **rs56337013 CYP2C19*5** | CCTCCTATGATTCACCGAACAGTTCTTGCATATTCTGTCTGTGCCAGTTATAGAGACAGTGTTTGTCACTCTCACAGTTACACATGAGGAGTAACTTCTCCCTATGTTTGTTATTTTCAGGAAAA**C**GGATTTGTGTGGGAGAGGGCCTGGCCCGCATGGAGCTGTTTTTATTCCTGACCTTCATTTTACAGAACTTTAACCTGAAATCTCTGATTGACCCAAAGGACCTTGACACAACTCCTGTTGTCAAT | CCTCCTATGATTCACCGAACAGTTCTTGCATATTCTGTCTGTGCCAGTTATAGAGACAGTGTTTGTCACTCTCACAGTTACACATGAGGAGTAACTTCTCCCTATGTTTGTTATTTTCAGGAAAA**T**GGATTTGTGTGGGAGAGGGCCTGGCCCGCATGGAGCTGTTTTTATTCCTGACCTTCATTTTACAGAACTTTAACCTGAAATCTCTGATTGACCCAAAGGACCTTGACACAACTCCTGTTGTCAAT | chr10:96612495 (GRCh37.p13) | B |
| **rs1799853 CYP2C9*2** | TCCTAGTTTCGTTTCTCTTCCTGTTAGGAATTGTTTTCAGCAATGGAAAGAAATGGAAGGAGATCCGGCGTTTCTCCCTCATGACGCTGCGGAATTTTGGGATGGGGAAGAGGAGCATTGAGGAC**C**GTGTTCAAGAGGAAGCCCGCTGCCTTGTGGAGGAGTTGAGAAAAACCAAGGGTGGGTGACCCTACTCCATATCACTGACCTTACTGGACTACTATCTTCTCTACTGACATTCTTGGAAACATTTC | TCCTAGTTTCGTTTCTCTTCCTGTTAGGAATTGTTTTCAGCAATGGAAAGAAATGGAAGGAGATCCGGCGTTTCTCCCTCATGACGCTGCGGAATTTTGGGATGGGGAAGAGGAGCATTGAGGAC**T**GTGTTCAAGAGGAAGCCCGCTGCCTTGTGGAGGAGTTGAGAAAAACCAAGGGTGGGTGACCCTACTCCATATCACTGACCTTACTGGACTACTATCTTCTCTACTGACATTCTTGGAAACATTTC | chr10:96702047 (GRCh37.p13) | B |
| **rs1057910 CYP2C9*3** | GTGTCTTATCAGCTAAAGTCCAGGAAGAGATTGAACGTGTGATTGGCAGAAACCGGAGCCCCTGCATGCAAGACAGGAGCCACATGCCCTACACAGATGCTGTGGTGCACGAGGTCCAGAGATAC**C**TTGACCTTCTCCCCACCAGCCTGCCCCATGCAGTGACCTGTGACATTAAATTCAGAAACTATCTCATTCCCAAGGTAAGTTTGTTTCTCCTACACTGCAACTCCATGTTTTCGAAGTCCCCAAAT | GTGTCTTATCAGCTAAAGTCCAGGAAGAGATTGAACGTGTGATTGGCAGAAACCGGAGCCCCTGCATGCAAGACAGGAGCCACATGCCCTACACAGATGCTGTGGTGCACGAGGTCCAGAGATAC**A**TTGACCTTCTCCCCACCAGCCTGCCCCATGCAGTGACCTGTGACATTAAATTCAGAAACTATCTCATTCCCAAGGTAAGTTTGTTTCTCCTACACTGCAACTCCATGTTTTCGAAGTCCCCAAAT | chr10:96741053 (GRCh37.p13) | B |
| **rs12248560 CYP2C19*17** | AGATAAATAAGTGGTTCTATTTAATGTGAAGCCTGTTTTATGAACAGGATGAATGTGGTATATATTCAGAATAACTAATGTTTGGAAGTTGTTTTGTTTTGCTAAAACAAAGTTTTAGCAAACGATTTTTTTTTTCAAATTTGTGTCTTCTGTTCTCAAAG**C**ATCTCTGATGTAAGAGATAATGCGCCACGATGGGCATCAGAAGACCTCAGCTCAAATCCCAGTTCTGCC | AGATAAATAAGTGGTTCTATTTAATGTGAAGCCTGTTTTATGAACAGGATGAATGTGGTATATATTCAGAATAACTAATGTTTGGAAGTTGTTTTGTTTTGCTAAAACAAAGTTTTAGCAAACGATTTTTTTTTTCAAATTTGTGTCTTCTGTTCTCAAAG**T**ATCTCTGATGTAAGAGATAATGCGCCACGATGGGCATCAGAAGACCTCAGCTCAAATCCCAGTTCTGCC | chr10:96521657 (GRCh37.p13) | B |
| **rs2231142 ABCG2** | GTTTTTCCACATTACCTTGGAGTCTGCCACTTTATCCAGACCTAACTCTTGAATGACCCTGTTAATCCGTTCGTTTTTTTCATGATTCGTCATAGTTGTTGCAAGCCGAAGAGCTGCTGAGAACT**G**TAAGTTTTCTCTCACCGTCAGAGTGCCCATCACAACATCATCCTTAAGGCAAATAGCATTTTAATGAGACATAATGATAATGAGTCTTTTCTAAGACCATGACTGTTTAGTATACATAACATAAT | GTTTTTCCACATTACCTTGGAGTCTGCCACTTTATCCAGACCTAACTCTTGAATGACCCTGTTAATCCGTTCGTTTTTTTCATGATTCGTCATAGTTGTTGCAAGCCGAAGAGCTGCTGAGAACT**T**TAAGTTTTCTCTCACCGTCAGAGTGCCCATCACAACATCATCCTTAAGGCAAATAGCATTTTAATGAGACATAATGATAATGAGTCTTTTCTAAGACCATGACTGTTTAGTATACATAACATAAT | chr4:89052323 (GRCh37.p13) | C |
| **rs1954787 GRIK4 c.83-10039T>C** | AGGTTGCAGTTAAGTCGTTGAGATGAAAGGACCGAAGTAGGGTGGTGGTGTTAAGACTGGAAAGAAGTGGACTGGTTTGAGAAATTTTGAGGAAGTACAACCAAAAGCAATTGGAGACTGGTTAT**C**GGAAGGTGCGGAATTGGGTGAAGGCACGATGCCTGGGTAGCTGGTGCTGCTATTAACTAAACGTAGGAATGTTAACAGGAAGAATCTAGGAGGGAAGAGGTGGGTTTACTTTGGGTCTTCCTAAA | AGGTTGCAGTTAAGTCGTTGAGATGAAAGGACCGAAGTAGGGTGGTGGTGTTAAGACTGGAAAGAAGTGGACTGGTTTGAGAAATTTTGAGGAAGTACAACCAAAAGCAATTGGAGACTGGTTAT**T**GGAAGGTGCGGAATTGGGTGAAGGCACGATGCCTGGGTAGCTGGTGCTGCTATTAACTAAACGTAGGAATGTTAACAGGAAGAATCTAGGAGGGAAGAGGTGGGTTTACTTTGGGTCTTCCTAAA | chr11:120663363 (GRCh37.p13) | C |
| **rs1799963 Factor 2** | AGAAGGTCATTGATCAGTTTGGAGAGTAGGGGGCCACTCATATTCTGGGCTCCTGGAACCAATCCCGTGAAAGAATTATTTTTGTGTTTCTAAAACTATGGTTCCCAATAAAAGTGACTCTCAGC**A**AGCCTCAATGCTCCCAGTGCTATTCATGGGCAGCTCTCTGGGCTCAGGAAGAGCCAGTAATACTACTGGATAAAGAAGACTTAAGAATCCACCACCTGGTGCACGCTGGTAGTCCGAGCACTCGG | AGAAGGTCATTGATCAGTTTGGAGAGTAGGGGGCCACTCATATTCTGGGCTCCTGGAACCAATCCCGTGAAAGAATTATTTTTGTGTTTCTAAAACTATGGTTCCCAATAAAAGTGACTCTCAGC**G**AGCCTCAATGCTCCCAGTGCTATTCATGGGCAGCTCTCTGGGCTCAGGAAGAGCCAGTAATACTACTGGATAAAGAAGACTTAAGAATCCACCACCTGGTGCACGCTGGTAGTCCGAGCACTCGG | chr11:46761055 (GRCh37.p13) | C |
| **rs505802 SLC22A12** | GGAGGAGTATTTGGACTGTGCCCATTCCACACAGTATGTGGCCTAAGGGTGAGCCTGGAGACAGCATTCCAGGCTGGAGGTCCAACATAAGCAAGGGTGGGAACTGAGAAAGCATGTACAGGGCA**C**GTGGTGGAAGCAGTGGATGACTCAAGGTGCAGAGCCCAGGGAGGCAAAGAAGTTGGCTTGGTAAGCCATGTGGAGGGGCAGCGGGAAGAGTCGGTGAAGCAAAAAGAAGCAGATGGGAGGGGCAT | GGAGGAGTATTTGGACTGTGCCCATTCCACACAGTATGTGGCCTAAGGGTGAGCCTGGAGACAGCATTCCAGGCTGGAGGTCCAACATAAGCAAGGGTGGGAACTGAGAAAGCATGTACAGGGCA**T**GTGGTGGAAGCAGTGGATGACTCAAGGTGCAGAGCCCAGGGAGGCAAAGAAGTTGGCTTGGTAAGCCATGTGGAGGGGCAGCGGGAAGAGTCGGTGAAGCAAAAAGAAGCAGATGGGAGGGGCAT | chr11:64357072 (GRCh37.p13) | C |
| **rs1135840 CYP2D6*2 (*39)** | CAGGGGACCCGAGTTGGAACTACCACATTGCTTTATTGTACATTAGAGCCTCTGGCTAGGGAGCAGGCTGGGGACTAGGTACCCCATTCTAGCGGGGCACAGCACAAAGCTCATAGGGGGATGGG**C**TCACCAGGAAAGCAAAGACACCATGGTGGCTGGGCCGGGGCTGTCCAGTGGGCACCGAGAAGCTGAAGTGCTGCAGCAGGGAGGTGAAGAAGAGGAAGAGCTCCATGCGGGCCAGGGGCTCCCCG | CAGGGGACCCGAGTTGGAACTACCACATTGCTTTATTGTACATTAGAGCCTCTGGCTAGGGAGCAGGCTGGGGACTAGGTACCCCATTCTAGCGGGGCACAGCACAAAGCTCATAGGGGGATGGG**G**TCACCAGGAAAGCAAAGACACCATGGTGGCTGGGCCGGGGCTGTCCAGTGGGCACCGAGAAGCTGAAGTGCTGCAGCAGGGAGGTGAAGAAGAGGAAGAGCTCCATGCGGGCCAGGGGCTCCCCG | chr22:42522613 (GRCh37.p13) | C |
| **rs28371706 CYP2D6*17** | CCACGACCCCGCGCCCTCTCTGCCCAGCTCGGACTACGGTCATCACCCACCCGGGTCCCACGGAAATCTGTCTCTGTCCCCACCGCTGCTTGCCTTGGGAACGCGGCCCGAAACCCAGGATCTGG**G**TGATGGGCACAGGCGGGCGGTCGGCGGTGTCCTCGCCGTGGGTCACCAGCGCCTCGCGCACGGCCGCCAGCCCATTGAGCACGACCACCGGCGTCCAGGCCAGCTGCAGGCTGAACACGTCCCCG | CCACGACCCCGCGCCCTCTCTGCCCAGCTCGGACTACGGTCATCACCCACCCGGGTCCCACGGAAATCTGTCTCTGTCCCCACCGCTGCTTGCCTTGGGAACGCGGCCCGAAACCCAGGATCTGG**A**TGATGGGCACAGGCGGGCGGTCGGCGGTGTCCTCGCCGTGGGTCACCAGCGCCTCGCGCACGGCCGCCAGCCCATTGAGCACGACCACCGGCGTCCAGGCCAGCTGCAGGCTGAACACGTCCCCG | chr22:42525772 (GRCh37.p13) | C |
| **rs28371725/rs16947 CYP2D6*41/CYP2D6*2 (*34)** | TCCGGCCCTGACACTCCTTCTTGCCTCCTATGTTGGAGGAGGTCAGGCTTACAGGATCCTGGTCAAGCCTGTGCTTGGAGCCCCGGGTGTCCCAGCAAAGTTCATGGGCCCCCGCCTGTACCCTT**C**CTCCCTCGGCCCCTGCACTGTTTCCCAGATGGGCTCACGCTGCACATCCGGATGTAGGATCATGAGCAGGAGGCCCCAGGCCAGCGTGGTCGAGGTGGTCACCATCCCGGCAGAGAACAGGTCAGCCACCACTATGC**A**CAGGTTCTCATCATTGAAGCTGCTCTCAGGGTTCCCCTTGGCCTGAGCAGGGCCGAGAGCATACTCGGGACAGAACGGGGTAGCCCCCAAATGACCTCCAATTCTGCACCTGTCAGCCCAGATGC | TCCGGCCCTGACACTCCTTCTTGCCTCCTATGTTGGAGGAGGTCAGGCTTACAGGATCCTGGTCAAGCCTGTGCTTGGAGCCCCGGGTGTCCCAGCAAAGTTCATGGGCCCCCGCCTGTACCCTT**T**CTCCCTCGGCCCCTGCACTGTTTCCCAGATGGGCTCACGCTGCACATCCGGATGTAGGATCATGAGCAGGAGGCCCCAGGCCAGCGTGGTCGAGGTGGTCACCATCCCGGCAGAGAACAGGTCAGCCACCACTATGC**G**CAGGTTCTCATCATTGAAGCTGCTCTCAGGGTTCCCCTTGGCCTGAGCAGGGCCGAGAGCATACTCGGGACAGAACGGGGTAGCCCCCAAATGACCTCCAATTCTGCACCTGTCAGCCCAGATGC | chr22:42523805/ chr22:42523943 (GRCh37.p13) | C |
| **rs35742686 CYP2D6*3** | TCCTCCTGGGACGCTCAACCCACCACCCTTGCCCCCCACCGTGGCAGCCACTCTCACCTTCTCCATCTCTGCCAGGAAGGCCTCAGTCAGGTCTCGGGGGGGCTGGGCTGGGTCCCAGGTCATCC**T**GTGCTCAGTTAGCAGCTCATCCAGCTGGGTCAGGAAAGCCTTTTGGAAGCGTAGGACCTTGCCAGCCAGCGCTGGGATATGCAGGAGGACGGGGACAGCATTCAGCACCTACACCAGACAGAACG | TCCTCCTGGGACGCTCAACCCACCACCCTTGCCCCCCACCGTGGCAGCCACTCTCACCTTCTCCATCTCTGCCAGGAAGGCCTCAGTCAGGTCTCGGGGGGGCTGGGCTGGGTCCCAGGTCATCC**-**GTGCTCAGTTAGCAGCTCATCCAGCTGGGTCAGGAAAGCCTTTTGGAAGCGTAGGACCTTGCCAGCCAGCGCTGGGATATGCAGGAGGACGGGGACAGCATTCAGCACCTACACCAGACAGAACGG | chr22:42524244 (GRCh37.p13) | C |
| **rs3892097/ rs5030655 CYP2D6*4/ CYP2D6*6** | CTGAGCTAGGTCCAGCAGCCTGAGGAAGCGAGGGTCGTCGTACTCGAAGCGGCGCCCGCAGGTGAGGGAGGCGATCACGTTGCTCACGGCTTTGTCCAAGAGACCGTTGGGGCGAAAGGGGCGTC**C**TGGGGGTGGGAGATGCGGGTAAGGGGTCGCCTTCCCCGTCCCCCGCCTTCCCAGTTCCCGCTTTGTGCCCTTCTGCCCATCACCCACCGGAGTGGTTGGCGAAGGCGGCACAAAGGCAGGCGGCCTCCTCGGTCACCC**A**CTGCTCCAGCGACTTCTTGCCCAGGCCCAAGTTGCGCAAGGTGGAGACGGAGAAGCGCCTCTGCTCGCGCCACGCGGGCCCATAGCGCGCCAGGAACACCCCTGGGGGTGGGACGGGCACGTGCG | CTGAGCTAGGTCCAGCAGCCTGAGGAAGCGAGGGTCGTCGTACTCGAAGCGGCGCCCGCAGGTGAGGGAGGCGATCACGTTGCTCACGGCTTTGTCCAAGAGACCGTTGGGGCGAAAGGGGCGTC**T**TGGGGGTGGGAGATGCGGGTAAGGGGTCGCCTTCCCCGTCCCCCGCCTTCCCAGTTCCCGCTTTGTGCCCTTCTGCCCATCACCCACCGGAGTGGTTGGCGAAGGCGGCACAAAGGCAGGCGGCCTCCTCGGTCACCC**-**CTGCTCCAGCGACTTCTTGCCCAGGCCCAAGTTGCGCAAGGTGGAGACGGAGAAGCGCCTCTGCTCGCGCCACGCGGGCCCATAGCGCGCCAGGAACACCCCTGGGGGTGGGACGGGCACGTGCGC | chr22:42524947/ chr22:42525086 (GRCh37.p13) | C |
| **rs1065852 CYP2D6*10** | GGCATCCTCAGGACCTCTGCCGCCCTCCAGGACCTCCTCCCTCACCTGGTCGAAGCAGTATGGTGTGTTCTGGAAGTCCACATGCAGCAGGTTGCCCAGCCCGGGCAGTGGCAGGGGGCCTGGTG**A**GTAGCGTGCAGCCCAGCGTTGGCGCCGGTGCATCAGGTCCACCAGGAGCAGGAAGATGGCCACTATCACGGCCAGGGGCACCAGTGCTTCTAGCCCCATACCTGCCTCACTACCAAATGGGCTCC | GGCATCCTCAGGACCTCTGCCGCCCTCCAGGACCTCCTCCCTCACCTGGTCGAAGCAGTATGGTGTGTTCTGGAAGTCCACATGCAGCAGGTTGCCCAGCCCGGGCAGTGGCAGGGGGCCTGGTG**G**GTAGCGTGCAGCCCAGCGTTGGCGCCGGTGCATCAGGTCCACCAGGAGCAGGAAGATGGCCACTATCACGGCCAGGGGCACCAGTGCTTCTAGCCCCATACCTGCCTCACTACCAAATGGGCTCC | chr22:42526694 (GRCh37.p13) | C |
| **rs1800462 TPMT*2** | AAATACTTTGGTTCCAGGAATTTCGGTGATTGGTTCTTCTGAGTAAGAAAGATTCTGCTCTGTAAAAAATTCTTGTATCCCAAGTTCACTGATTTCCACACCAACTACACTGTGTCCCCGGTCTG**C**AAACCTGCATAAAATCATACATTTACACTTAAATTATGTTTTCAAATGACTAAATAGAGGGTTATATTAGAGTAAGCATATATTTTCTTTAATTTAGAGGAATTTATATGAATTCAGGTTCATAG | AAATACTTTGGTTCCAGGAATTTCGGTGATTGGTTCTTCTGAGTAAGAAAGATTCTGCTCTGTAAAAAATTCTTGTATCCCAAGTTCACTGATTTCCACACCAACTACACTGTGTCCCCGGTCTG**G**AAACCTGCATAAAATCATACATTTACACTTAAATTATGTTTTCAAATGACTAAATAGAGGGTTATATTAGAGTAAGCATATATTTTCTTTAATTTAGAGGAATTTATATGAATTCAGGTTCATAG | chr6:18143955 (GRCh37.p13) | D |
| **rs1183201 SLC17A1** | TAATTATCTTCAGTTTTATCTATGTTGTTCTTTGTGTCAGTAGTTCATTCCATTATACTGCTAAGTAGTACTCCATTTTATGGATGTGCCATAGTTTGTTCAATCATTCACCTACTGATGGACAT**A**TAGGTTATTTCCAGTTTTTTGGCTATCAGAAATAAAGCTAGACAAAAATGTTTATAGAACATTTATGTACAAGTCTCTGAATGGACATATGCTTTTATTTCTCTTCAGTAAATAACTAGCAGTGG | TAATTATCTTCAGTTTTATCTATGTTGTTCTTTGTGTCAGTAGTTCATTCCATTATACTGCTAAGTAGTACTCCATTTTATGGATGTGCCATAGTTTGTTCAATCATTCACCTACTGATGGACAT**T**TAGGTTATTTCCAGTTTTTTGGCTATCAGAAATAAAGCTAGACAAAAATGTTTATAGAACATTTATGTACAAGTCTCTGAATGGACATATGCTTTTATTTCTCTTCAGTAAATAACTAGCAGTGG | chr6:25823444 (GRCh37.p13) | D |
| **rs1799945 HFE H63D** | TTGCTCTGTCTCCAGGTTCACACTCTCTGCACTACCTCTTCATGGGTGCCTCAGAGCAGGACCTTGGTCTTTCCTTGTTTGAAGCTTTGGGCTACGTGGATGACCAGCTGTTCGTGTTCTATGAT**C**ATGAGAGTCGCCGTGTGGAGCCCCGAACTCCATGGGTTTCCAGTAGAATTTCAAGCCAGATGTGGCTGCAGCTGAGTCAGAGTCTGAAAGGGTGGGATCACATGTTCACTGTTGACTTCTGGACT | TTGCTCTGTCTCCAGGTTCACACTCTCTGCACTACCTCTTCATGGGTGCCTCAGAGCAGGACCTTGGTCTTTCCTTGTTTGAAGCTTTGGGCTACGTGGATGACCAGCTGTTCGTGTTCTATGAT**G**ATGAGAGTCGCCGTGTGGAGCCCCGAACTCCATGGGTTTCCAGTAGAATTTCAAGCCAGATGTGGCTGCAGCTGAGTCAGAGTCTGAAAGGGTGGGATCACATGTTCACTGTTGACTTCTGGACT | chr6:26091179 (GRCh37.p13) | D |
| **rs1800562 HFE C282Y** | GAAGGATAAGCAGCCAATGGATGCCAAGGAGTTCGAACCTAAAGACGTATTGCCCAATGGGGATGGGACCTACCAGGGCTGGATAACCTTGGCTGTACCCCCTGGGGAAGAGCAGAGATATACGT**G**CCAGGTGGAGCACCCAGGCCTGGATCAGCCCCTCATTGTGATCTGGGGTATGTGACTGATGAGAGCCAGGAGCTGAGAAAATCTATTGGGGGTTGAGAGGAGTGCCTGAGGAGGTAATTATGGCA | GAAGGATAAGCAGCCAATGGATGCCAAGGAGTTCGAACCTAAAGACGTATTGCCCAATGGGGATGGGACCTACCAGGGCTGGATAACCTTGGCTGTACCCCCTGGGGAAGAGCAGAGATATACGT**A**CCAGGTGGAGCACCCAGGCCTGGATCAGCCCCTCATTGTGATCTGGGGTATGTGACTGATGAGAGCCAGGAGCTGAGAAAATCTATTGGGGGTTGAGAGGAGTGCCTGAGGAGGTAATTATGGCA | chr6:26093141 (GRCh37.p13) | D |
| **rs1142345/ rs1800584 TPMT*3C/ TPMT*4** | ATTTATAAACAATTTTAAAAATTCATCCATTACATTTTCAGGCTTTAGCATAATTTTCAATTCCTCAAAAACATGTCAGTGTGATTTTATTTTATCTATGTCTCATTTACTTTTCTGTAAGTAGA**C**ATAACTTTTCAAAAAGACAGTCAATTCCCCAACTTTTATGTCGTTCTTCAAAAGCATCAACCTTCTCAAGACAACGTATATTGCATATTTTAC**C**TGAAACAAGAAAGAGTAACATGTTAAAATACTATGAAGAATGACATCAGGGATTCTTTTAAAAATACTCAAAATTGGCTGGGTGCGGTGGCTCACACCTGTAATCCCAACACTTTGGGAGGCCGA | ATTTATAAACAATTTTAAAAATTCATCCATTACATTTTCAGGCTTTAGCATAATTTTCAATTCCTCAAAAACATGTCAGTGTGATTTTATTTTATCTATGTCTCATTTACTTTTCTGTAAGTAGA**T**ATAACTTTTCAAAAAGACAGTCAATTCCCCAACTTTTATGTCGTTCTTCAAAAGCATCAACCTTCTCAAGACAACGTATATTGCATATTTTAC**T**TGAAACAAGAAAGAGTAACATGTTAAAATACTATGAAGAATGACATCAGGGATTCTTTTAAAAATACTCAAAATTGGCTGGGTGCGGTGGCTCACACCTGTAATCCCAACACTTTGGGAGGCCGA | chr6:18130918/ chr6:18131012 (GRCh37.p13) | D |
| **rs1800460 TPMT*3B** | ATAGAAGTCTAAGCTGATTTTCTAGAACCCAGAAAAAGTATAGTATACTAAAAAATTAAGACAGCTAAACAAAAAAAGAAAAATTACTTACCATTTGCGATCACCTGGATTGATGGCAACTAATG**T**TCCTCTATCCCAAATCATGTCAAATTTGCCAATATTTGTCCTACCAGAAAGAGAAAAAACATTTTATGGGAGAAAAATCAAATCTTTAAGAAGATGAGCAGCGTCCCCCATGGTGCATGCTGGTA | ATAGAAGTCTAAGCTGATTTTCTAGAACCCAGAAAAAGTATAGTATACTAAAAAATTAAGACAGCTAAACAAAAAAAGAAAAATTACTTACCATTTGCGATCACCTGGATTGATGGCAACTAATG**C**TCCTCTATCCCAAATCATGTCAAATTTGCCAATATTTGTCCTACCAGAAAGAGAAAAAACATTTTATGGGAGAAAAATCAAATCTTTAAGAAGATGAGCAGCGTCCCCCATGGTGCATGCTGGTA | chr6:18139228 (GRCh37.p13) | D |
| **rs776746 CYP3A5*3** | TGTACGACACACAGCAACCTTAGGTTCTAGTTCATTAGGGTGTGACACACAGCAAGAGTCTCACACAGGAGCCACCCAAGGCTTCATATGATGAAGGGTAATGTGGTCCAAACAGGGAAGAGATA**T**TGAAAGACAAAAGAGCTCTTTAAAGAGATTATGGTTAGAAATGACAGTAGAGCATTCGTTAAGCTGGGTGGTACATACGTGGGTATCTCCTATGCCACTCTCCATAATGTTTTATATGTTTAAAA | TGTACGACACACAGCAACCTTAGGTTCTAGTTCATTAGGGTGTGACACACAGCAAGAGTCTCACACAGGAGCCACCCAAGGCTTCATATGATGAAGGGTAATGTGGTCCAAACAGGGAAGAGATA**C**TGAAAGACAAAAGAGCTCTTTAAAGAGATTATGGTTAGAAATGACAGTAGAGCATTCGTTAAGCTGGGTGGTACATACGTGGGTATCTCCTATGCCACTCTCCATAATGTTTTATATGTTTAAAA | chr7:99270539 (GRCh37.p13) | D |
| **rs1050828 G6PD*Asahi** | CATGCTGGGGGCTGGTAGAGAGGGCAGAACCAGGCTGGGGGAGGCCCTGACACCACCCACCTTGAAGAAGGGCTCACTCTGTTTGCGGATGTCAGCCACTGTGAGGCGGGAACGGGCATAGCCCA**C**GATGAAGGTGTTTTCGGGCAGAAGGCCATCCCGGAACAGCCACCTGAGGGCAGGGCACAGCTGTAACCAGTGCGGGCAGGGCAGGACCAGGCCTGTCCCTGGCGGGAGGTCACAGGGGCAGTGGT | CATGCTGGGGGCTGGTAGAGAGGGCAGAACCAGGCTGGGGGAGGCCCTGACACCACCCACCTTGAAGAAGGGCTCACTCTGTTTGCGGATGTCAGCCACTGTGAGGCGGGAACGGGCATAGCCCA**T**GATGAAGGTGTTTTCGGGCAGAAGGCCATCCCGGAACAGCCACCTGAGGGCAGGGCACAGCTGTAACCAGTGCGGGCAGGGCAGGACCAGGCCTGTCCCTGGCGGGAGGTCACAGGGGCAGTGGT | chrX:153764217 (GRCh37.p13) | D |
| **rs72554665 G6PD* Bangkok Noi** | CCCTGCCCGCTGGGCTCTGTCCCCAGCCCCCACCCTTTCCTCACCTGCCATAAATATAGGGGATGGGCTTGGGCTTCTCCAGCTCAATCTGGTGCAGCAGTGGGGTGAAAATACGCCAGGCCTCA**C**GGAGCTCGTCGCTGAGGGGACATAGTATGGCTTGGGAGGCCGGTGGCACACAGGGAGGGAGGGCAAAGGCCACCCCATAGCCCACAGGTATGCAGGGGCCGGCAGCTGGGCCTCACCTGCGCACG | CCCTGCCCGCTGGGCTCTGTCCCCAGCCCCCACCCTTTCCTCACCTGCCATAAATATAGGGGATGGGCTTGGGCTTCTCCAGCTCAATCTGGTGCAGCAGTGGGGTGAAAATACGCCAGGCCTCA**A**GGAGCTCGTCGCTGAGGGGACATAGTATGGCTTGGGAGGCCGGTGGCACACAGGGAGGGAGGGCAAAGGCCACCCCATAGCCCACAGGTATGCAGGGGCCGGCAGCTGGGCCTCACCTGCGCACG | chrX:153760484 (GRCh37.p13) | D |
| **rs5030868 G6PD* Mediterranean** | AGTACCACCCCCACCCTGGTCCCCCGGCCCAGGCTTGGCCCCACCTCAGCACCATGAGGTTCTGCACCATCTCCTTGCCCAGGTAGTGGTCGATGCGGTAGATCTGGTCCTCACGGAACAGGGAG**A**AGATGTGGTTGGACAGCCGGTCAGAGCTCTGCAGGTCCCTCCCGAAGGGCTTCTCCACGATGATGCGGTTCCAGCCTCTGCTGGGAGCCCGGAGCTGCGTTACCCCCTTGAACCCCTCTTCGGGG | AGTACCACCCCCACCCTGGTCCCCCGGCCCAGGCTTGGCCCCACCTCAGCACCATGAGGTTCTGCACCATCTCCTTGCCCAGGTAGTGGTCGATGCGGTAGATCTGGTCCTCACGGAACAGGGAG**G**AGATGTGGTTGGACAGCCGGTCAGAGCTCTGCAGGTCCCTCCCGAAGGGCTTCTCCACGATGATGCGGTTCCAGCCTCTGCTGGGAGCCCGGAGCTGCGTTACCCCCTTGAACCCCTCTTCGGGG | chrX:153762634 (GRCh37.p13) | D |

**Supplemental Table 3. gBlocks for each synthetic control in Version 1.2 Alterations are the highlighted base. VIC probes will bind to bases highlighted in green. FAM probes will bind to bases highlighted in red. Rows highlighted in yellow indicate 2 tested SNPs being within 251bp of one another.**

| **NCBI SNP Reference** | **VIC SNP Sequence** | **FAM SNP Sequence** | **Genomic Location** | **"Stitched" Fragment** |
| --- | --- | --- | --- | --- |
| **rs7294 VKORC1*3** | TTGCTCAGAACCTTCCCTCCCTGGGCAATGGAAAGAGCTTTGGAGACCAGCCCATGGGGACAGAGTCAGAGGCACTGGGTGTAAAAAAGAGCGAGCGTGTGGCACATTTGGTCCATTGTCATGTG**C**GGGTATGGCAGGAGGAGGGGGTAATCTAGAAGCCCCACATCTAGGGCCTTCTAGGGACCCAGATATGCCCCCTTAGGCAAGGCTCACATGCCAAAGCAAAGCAGATGAGGTCAGCCTGGCTTGGG | NA | chr16:31102321 (GRCh37.p13) | VIC22 |
| **rs7294 VKORC1*3** | NA | TTGCTCAGAACCTTCCCTCCCTGGGCAATGGAAAGAGCTTTGGAGACCAGCCCATGGGGACAGAGTCAGAGGCACTGGGTGTAAAAAAGAGCGAGCGTGTGGCACATTTGGTCCATTGTCATGTG**T**GGGTATGGCAGGAGGAGGGGGTAATCTAGAAGCCCCACATCTAGGGCCTTCTAGGGACCCAGATATGCCCCCTTAGGCAAGGCTCACATGCCAAAGCAAAGCAGATGAGGTCAGCCTGGCTTGGG | chr16:31102321 (GRCh37.p13) | FAM22 |
| **rs5030655 CYP2D6*6** | CTGAGCTAGGTCCAGCAGCCTGAGGAAGCGAGGGTCGTCGTACTCGAAGCGGCGCCCGCAGGTGAGGGAGGCGATCACGTTGCTCACGGCTTTGTCCAAGAGACCGTTGGGGCGAAAGGGGCGTCCTGGGGGTGGGAGATGCGGGTAAGGGGTCGCCTTCCCCGTCCCCCGCCTTCCCAGTTCCCGCTTTGTGCCCTTCTGCCCATCACCCACCGGAGTGGTTGGCGAAGGCGGCACAAAGGCAGGCGGCCTCCTCGGTCACCC**A**CTGCTCCAGCGACTTCTTGCCCAGGCCCAAGTTGCGCAAGGTGGAGACGGAGAAGCGCCTCTGCTCGCGCCACGCGGGCCCATAGCGCGCCAGGAACACCCCTGGGGGTGGGACGGGCACGTGCG | NA | chr22:42525086 (GRCh37.p13) | VIC39 |
| **rs5030655 CYP2D6*6** | NA | CTGAGCTAGGTCCAGCAGCCTGAGGAAGCGAGGGTCGTCGTACTCGAAGCGGCGCCCGCAGGTGAGGGAGGCGATCACGTTGCTCACGGCTTTGTCCAAGAGACCGTTGGGGCGAAAGGGGCGTCTTGGGGGTGGGAGATGCGGGTAAGGGGTCGCCTTCCCCGTCCCCCGCCTTCCCAGTTCCCGCTTTGTGCCCTTCTGCCCATCACCCACCGGAGTGGTTGGCGAAGGCGGCACAAAGGCAGGCGGCCTCCTCGGTCACCC**-**TGCTCCAGCGACTTCTTGCCCAGGCCCAAGTTGCGCAAGGTGGAGACGGAGAAGCGCCTCTGCTCGCGCCACGCGGGCCCATAGCGCGCCAGGAACACCCCTGGGGGTGGGACGGGCACGTGCGC | chr22:42525086 (GRCh37.p13) | FAM39 |
| **rs9332131 CYP2C9*6** | ACAAATTACTTAAAAACGTTGCTTTTATGAAAAGTTATATTTTGGAAAAAGTAAAAGAACACCAAGAATCAATGGACATGAACAACCCTCAGGACTTTATTGATTGCTTCCTGATGAAAATGGAG**-**GGTAAAATGTAAACAAAAGCTTAGTTATGTGACTGCTTGTGAATTTGTGATTTGTTGACTAGTTCTGTGTTTACTAAGGATGTTTAACTGGTCAATCAGTAATGCTTGAGAAGCACTTTAAGTT | ACAAATTACTTAAAAACGTTGCTTTTATGAAAAGTTATATTTTGGAAAAAGTAAAAGAACACCAAGAATCAATGGACATGAACAACCCTCAGGACTTTATTGATTGCTTCCTGATGAAAATGGAG**A**AGGTAAAATGTAAACAAAAGCTTAGTTATGTGACTGCTTGTGAATTTGTGATTTGTTGACTAGTTCTGTGTTTACTAAGGATGTTTAACTGGTCAATCAGTAATGCTTGAGAAGCACTTTAAGTT | chr10: 96709040 (GRCh37.p13) | E |
| **rs28371685/ rs28371686 CYP2C9*11/ CYP2C9*5** | TTCATGATTCATATACCCCTGAATTGCTACAACAAATGTGCCATTTTTCTCCTTTTCCATCAGTTTTTACTTGTGTCTTATCAGCTAAAGTCCAGGAAGAGATTGAACGTGTGATTGGCAGAAAC**T**GGAGCCCCTGCATGCAAGACAGGAGCCACATGCCCTACACAGATGCTGTGGTGCACGAGGTCCAGAGATACATTGA**C**CTTCTCCCCACCAGCCTGCCCCATGCAGTGACCTGTGACATTAAATTCAGAAACTATCTCATTCCCAAGGTAAGTTTGTTTCTCCTACACTGCAACTCCATGTTTTCGAAGTCCCCAAATTCATA | TTCATGATTCATATACCCCTGAATTGCTACAACAAATGTGCCATTTTTCTCCTTTTCCATCAGTTTTTACTTGTGTCTTATCAGCTAAAGTCCAGGAAGAGATTGAACGTGTGATTGGCAGAAAC**C**GGAGCCCCTGCATGCAAGACAGGAGCCACATGCCCTACACAGATGCTGTGGTGCACGAGGTCCAGAGATACATTGA**G**CTTCTCCCCACCAGCCTGCCCCATGCAGTGACCTGTGACATTAAATTCAGAAACTATCTCATTCCCAAGGTAAGTTTGTTTCTCCTACACTGCAACTCCATGTTTTCGAAGTCCCCAAATTCATA | chr10: 96740981/chr10: 96741058 (GRCh37.p13) | E |
| **rs7900194 CYP2C9*8** | TCTCCCTCCTAGTTTCGTTTCTCTTCCTGTTAGGAATTGTTTTCAGCAATGGAAAGAAATGGAAGGAGATCCGGCGTTTCTCCCTCATGACGCTGCGGAATTTTGGGATGGGGAAGAGGAGCATTGAGGACCGTGTTCAAGAGGAAGCCC**A**CTGCCTTGTGGAGGAGTTGAGAAAAACCAAGGGTGGGTGACCCTACTCCATATCACTGACCTTACTGGACTACTATCTTCTCTACTGACATTCTTGGAAACATTTCAGGGGTGGCCATATCTTTCATTATGAGTCCTGGTTGTTAGCTC | TCTCCCTCCTAGTTTCGTTTCTCTTCCTGTTAGGAATTGTTTTCAGCAATGGAAAGAAATGGAAGGAGATCCGGCGTTTCTCCCTCATGACGCTGCGGAATTTTGGGATGGGGAAGAGGAGCATTGAGGACCGTGTTCAAGAGGAAGCCC**G**CTGCCTTGTGGAGGAGTTGAGAAAAACCAAGGGTGGGTGACCCTACTCCATATCACTGACCTTACTGGACTACTATCTTCTCTACTGACATTCTTGGAAACATTTCAGGGGTGGCCATATCTTTCATTATGAGTCCTGGTTGTTAGCTC | chr10: 96702066 (GRCh37.p13) | F |

Supplemental Tables 2 and 3: Due to performance issues of specific SNPs in the initial build, the location of SNPs to other assayed SNPs, and the addition of more SNPs to the original PGx panel, 8 additional synthetic controls were designed and synthesized (Version 1.2). Three of Version 1.2 synthetic controls (“VIC22”, “FAM22”, and “VIC39”) could be pooled into the original “VIC” and “FAM” and “VICFAM” pools without causing interference. However, 5 controls could not be pooled into the aforementioned pools without causing interference. Thus, 4 additional control pools denoted “FAM39”, “VIC_EF”, “FAM_EF” and “VICFAM_EF” are included and validated in this assay.

**Supplemental Table 4. TaqMan CNV Assay Overview for CYP2D6**

| **Assay Name** | **Gene** | **Intron/Exon** | **Probe Type** | **ThermoFisher Assay ID** |
| --- | --- | --- | --- | --- |
| RNaseP | RNaseP | Not Listed | FAM-TAMRA | 4316838 |
| CYP2D6 19B03 Exon9 | CYP2D6 | Exon 9 | FAM-MGB | Hs00010001_cn |
| CYP2D6 19B04 Intron 6 | CYP2D6 | Intron 6 | FAM-MGB | Hs04502391_cn |
| CYP2D6 19B05 Intron 2 | CYP2D6 | Intron 2 | FAM-MGB | Hs04083572_cn |

**Supplemental Table 5. Subset of Get-RM Pharmacogenomics Results Summary**

| **Coriell DNA Ref** | **Coriell CC Ref** | **CYP2D6** | **CYP2C19** | **CYP2C9** | **VKORC1^#^** | **UGT1A1** |
| --- | --- | --- | --- | --- | --- | --- |
| [NA02016](http://ccr.coriell.org/Sections/Search/Sample_Detail.aspx?Ref=GM02016&PgId=166) | [GM02016](https://catalog.coriell.org/0/Sections/Search/Sample_Detail.aspx?Ref=GM02016&product=CC) | *2XN/*17 | *1/*2 | *1/*1 | WT/-1639G>A | *1/*1 |
| [NA07439](http://ccr.coriell.org/Sections/Search/Sample_Detail.aspx?Ref=GM07439&PgId=166) | [GM07439](https://catalog.coriell.org/0/Sections/Search/Sample_Detail.aspx?Ref=GM07439&product=CC) | *4XN/*41 | *2/*2 (*2/*10) | *1/*1 (*1/*9) | WT/WT | *1/*28 |
| [NA12244](http://ccr.coriell.org/Sections/Search/Sample_Detail.aspx?Ref=GM12244&PgId=166) | [GM12244](https://catalog.coriell.org/0/Sections/Search/Sample_Detail.aspx?Ref=GM12244&product=CC) | *35/*41 | *1/*1 | *2/*3 | WT/WT | *1/*28 |
| [NA16654](http://ccr.coriell.org/Sections/Search/Sample_Detail.aspx?Ref=GM16654&PgId=166) | [GM16654](https://catalog.coriell.org/0/Sections/Search/Sample_Detail.aspx?Ref=GM16654&product=CC) | *10/*10 | *1/*2 | *1/*1 | WT/-1639G>A | *1/*1 |
| [NA16688](http://ccr.coriell.org/Sections/Search/Sample_Detail.aspx?Ref=GM16688&PgId=166) | [GM16688](https://catalog.coriell.org/0/Sections/Search/Sample_Detail.aspx?Ref=GM16688&product=CC) | *2/*10 | *2/*3 | *1/*1 | -1639G>A/-1639G>A | *1/*1 |
| [NA17019](http://ccr.coriell.org/Sections/Search/Sample_Detail.aspx?Ref=GM17019&PgId=166) | [GM17019](https://catalog.coriell.org/0/Sections/Search/Sample_Detail.aspx?Ref=GM17019&product=CC) | *1/*10 | *1/*1 | *1/*3 | WT/-1639G>A | *1/*1 |
| [NA17039](http://ccr.coriell.org/Sections/Search/Sample_Detail.aspx?Ref=GM17039&PgId=166) | [GM17039](https://catalog.coriell.org/0/Sections/Search/Sample_Detail.aspx?Ref=GM17039&product=CC) | *2/*17 | *1/*1 | *1/*1 (*1/*9) | WT/WT | *1/*36 |
| [NA17075](http://ccr.coriell.org/Sections/Search/Sample_Detail.aspx?Ref=GM17075&PgId=166) | [GM17075](https://catalog.coriell.org/0/Sections/Search/Sample_Detail.aspx?Ref=GM17075&product=CC) | *1/*2 | *1/*1 | *2/*2 | WT/-1639G>A | *1/*28 |
| [NA17084](http://ccr.coriell.org/Sections/Search/Sample_Detail.aspx?Ref=GM17084&PgId=166) | [GM17084](https://catalog.coriell.org/0/Sections/Search/Sample_Detail.aspx?Ref=GM17084&product=CC) | *1/*10 | *2/*4 | *1/*2 | WT/-1639G>A | *1/*1 |
| [NA17119](http://ccr.coriell.org/Sections/Search/Sample_Detail.aspx?Ref=GM17119&PgId=166) | [GM17119](https://catalog.coriell.org/0/Sections/Search/Sample_Detail.aspx?Ref=GM17119&product=CC) | *1/*2 | *1/*1 (*1/*17) | *1/*1 | WT/WT | *1/*37 |
| [NA17129](http://ccr.coriell.org/Sections/Search/Sample_Detail.aspx?Ref=GM17129&PgId=166) | [GM17129](https://catalog.coriell.org/0/Sections/Search/Sample_Detail.aspx?Ref=GM17129&product=CC) | *1/*4 | *1/*1 | *1/*2 | WT/WT | *1/*36 |
| [NA17205](http://ccr.coriell.org/Sections/Search/Sample_Detail.aspx?Ref=GM17205) | [GM17205](https://catalog.coriell.org/0/Sections/Search/Sample_Detail.aspx?Ref=GM17205&product=CC) | *1/*41 | *1/*2 | *1/*2 | WT/-1639G>A | *1/*1 |
| [NA17221](http://ccr.coriell.org/Sections/Search/Sample_Detail.aspx?Ref=GM17221&PgId=166) | [GM17221](https://catalog.coriell.org/0/Sections/Search/Sample_Detail.aspx?Ref=GM17221&product=CC) | *1XN/*2 | *1/*1 | *2/*3 | WT/-1639G>A | *1/*1 |
| [NA17232](http://ccr.coriell.org/Sections/Search/Sample_Detail.aspx?Ref=GM17232) | [GM17232](https://catalog.coriell.org/0/Sections/Search/Sample_Detail.aspx?Ref=GM17232&product=CC) | *2/*2XN | *1/*2 (*2/*17) | *1/*1 | WT/-1639G>A | *1/*1 |
| [NA17244](http://ccr.coriell.org/Sections/Search/Sample_Detail.aspx?Ref=GM17244) | [GM17244](https://catalog.coriell.org/0/Sections/Search/Sample_Detail.aspx?Ref=GM17244&product=CC) | DUP/*4/*2A | *1/*1 | *1/*1 | -1639G>A/-1639G>A | *1/*1 |
| [NA17246](http://ccr.coriell.org/Sections/Search/Sample_Detail.aspx?Ref=GM17246&PgId=166) | [GM17246](https://catalog.coriell.org/0/Sections/Search/Sample_Detail.aspx?Ref=GM17246&product=CC) | *4/*35 | *1/*8 (*8/*17) | *1/*2 | WT/-1639G>A | *1/*28 |
| [NA17252](http://ccr.coriell.org/Sections/Search/Sample_Detail.aspx?Ref=GM17252&PgId=166) | [GM17252](https://catalog.coriell.org/0/Sections/Search/Sample_Detail.aspx?Ref=GM17252&product=CC) | *4/*5 | *1/*1 | *2/*3 | WT/-1639G>A | *28/*28 |
| [NA17279](http://ccr.coriell.org/Sections/Search/Sample_Detail.aspx?Ref=GM17279) | [GM17279](https://catalog.coriell.org/0/Sections/Search/Sample_Detail.aspx?Ref=GM17279&product=CC) | *4/*41 | *1/*1 (*17/*17) | *1/*1 | WT/WT | *1/*1 |
| [NA17280](http://ccr.coriell.org/Sections/Search/Sample_Detail.aspx?Ref=GM17280&PgId=166) | [GM17280](https://catalog.coriell.org/0/Sections/Search/Sample_Detail.aspx?Ref=GM17280&product=CC) | *2/*3 | *1/*8 | *1/*2 | WT/WT | *1/*1 |
| [NA17298](http://ccr.coriell.org/Sections/Search/Sample_Detail.aspx?Ref=GM17298&PgId=166) | [GM17298](https://catalog.coriell.org/0/Sections/Search/Sample_Detail.aspx?Ref=GM17298&product=CC) | *1/*1XN | *1/*1 | *1/*1 | WT/-1639G>A | *1/*28 |
| [NA17300](http://ccr.coriell.org/Sections/Search/Sample_Detail.aspx?Ref=GM17300&PgId=166) | [GM17300](https://catalog.coriell.org/0/Sections/Search/Sample_Detail.aspx?Ref=GM17300&product=CC) | *1/*6 | *1/*1 (*1/*17) | *1/*1 | WT/-1639G>A | *1/*28 |

**Supplemental Table 6. SNP Comparisons. SNPs in bold denote SNPs compared for that particular sample. The letter n is the number of clinical samples compared.**

|  | **PCR-RFLP; n=88** | **NGS; n=34** | **Indiana Blind; n=10** | **Genomind, Inc. Blind; n=4** | **Coriell Get-RM PGx; n=21** | **Coriell 1000Genomes; n=3** | **Sendouts; n=8** | **Sanger; n=6** |
| --- | --- | --- | --- | --- | --- | --- | --- | --- |
| **ABCG2** | ABCG2 | ***ABCG2*** | ABCG2 | ABCG2 | ABCG2 | ***ABCG2*** | ABCG2 | ABCG2 |
| **CYP2B6** | CYP2B6*9 | CYP2B6*9 | CYP2B6*9 | CYP2B6*9 | CYP2B6*9 | ***CYP2B6*9*** | ***CYP2B6*9*** | ***CYP2B6*9*** |
| **CYP2C19** | CYP2C19*2,*3,*4,*5,*6,*8,*17 | ***CYP2C19*2,*3,*4,*5,*6,*8***,*17 | ***CYP2C19*2,*3,*4,****5,****6,*8,*17*** | ***CYP2C19****2,*3,*4,****5,*6***,*8,*17 | ***CYP2C19*2,*3,*4,*5,*6,*8,*17*** | ***CYP2C19*2,*3,*4,*5,*6,*8,*17*** | ***CYP2C19*2,*3,*4,*5,*6,*8,*17*** | CYP2C19*2,*3,*4,*5,*6,*8,*17 |
| **CYP2C9** | CYP2C9*2,*3,*5,*6,*8,*11 | ***CYP2C9*2,*3,*5,*6,****8,****11*** | ***CYP2C9*2,*3,*5,*6,*8,*11*** | CYP2C9*2,*3,*5,*6,*8,*11 | ***CYP2C9*2,*3,*5,*6,*8,*11*** | ***CYP2C9*2,*3,*5,*6,*8,*11*** | ***CYP2C9*2,*3,*5,*6***,*8,*11 | CYP2C9*2,*3,*5,*6,*8,*11 |
| **CYP2D6** | CYP2D6*2(*34),*2(*39),*3,*4,*6,*10,*17,*41 | ***CYP2D6*2(*34),*2(*39),*3,*4,*6,*10,*17,*41*** | ***CYP2D6*2(*34),*2(*39),*3,*4,*6,*10,*17,*41*** | CYP2D6*2(*34),*2(*39),*3,*4,*6,*10,*17,*41 | ***CYP2D6*2(*34),*2(*39),*3,*4,*6,*10,*17,*41*** | ***CYP2D6*2(*34),*2(*39),*3,*4,*6,*10,*17,*41*** | ***CYP2D6*2(*34),*2(*39),*3,*4,*6,*10,*17,*41*** | CYP2D6*2(*34),*2(*39),*3,*4,*6,*10,*17,*41 |
| **CYP3A5** | CYP3A5*3 | ***CYP3A5*3*** | ***CYP3A5*3*** | CYP3A5*3 | CYP3A5*3 | ***CYP3A5*3*** | CYP3A5*3 | CYP3A5*3 |
| **CYP4F2** | CYP4F2*3 | ***CYP4F2*3*** | ***CYP4F2*3*** | CYP4F2*3 | CYP4F2*3 | ***CYP4F2*3*** | CYP4F2*3 | CYP4F2*3 |
| **DPYD** | DPYD D949V,*2A,*13 | ***DPYD D949V,*2A,*13*** | ***DPYD*** D949V,****2A***,*13 | DPYD D949V,*2A,*13 | DPYD D949V,*2A,*13 | ***DPYD D949V,*2A,*13*** | DPYD D949V,*2A,*13 | DPYD D949V,*2A,*13 |
| **Factor 2** | ***Factor 2*** | ***Factor 2*** | Factor 2 | Factor 2 | Factor 2 | ***Factor 2*** | Factor 2 | Factor 2 |
| **Factor 5** | ***Factor 5*** | ***Factor 5*** | Factor 5 | Factor 5 | Factor 5 | ***Factor 5*** | Factor 5 | Factor 5 |
| **G6PD** | G6PD*Asahi, *Bangkok Noi,*Mediterranean | ***G6PD*Asahi, *Bangkok Noi,*Mediterranean*** | ***G6PD*Asahi***, *Bangkok Noi,*Mediterranean | G6PD*Asahi, *Bangkok Noi,*Mediterranean | G6PD*Asahi, *Bangkok Noi,*Mediterranean | ***G6PD*Asahi, *Bangkok Noi,*Mediterranean*** | G6PD*Asahi, *Bangkok Noi,*Mediterranean | G6PD*Asahi, *Bangkok Noi,*Mediterranean |
| **GRIK4** | GRIK4 | GRIK4 | GRIK4 | GRIK4 | GRIK4 | ***GRIK4*** | GRIK4 | ***GRIK4*** |
| **HFE** | ***HFE H63D,C282Y*** | ***HFE H63D,C282Y*** | HFE H63D,C282Y | HFE H63D,C282Y | HFE H63D,C282Y | ***HFE H63D,C282Y*** | HFE H63D,C282Y | HFE H63D,C282Y |
| **MTHFR** | ***MTHFR*** | ***MTHFR*** | ***MTHFR*** | MTHFR | MTHFR | ***MTHFR*** | ***MTHFR*** | MTHFR |
| **SLC17A1** | SLC17A1 | SLC17A1 | SLC17A1 | SLC17A1 | SLC17A1 | ***SLC17A1*** | SLC17A1 | ***SLC17A1*** |
| **SLC22A12** | SLC22A12 | SLC22A12 | SLC22A12 | SLC22A12 | SLC22A12 | ***SLC22A12*** | SLC22A12 | ***SLC22A12*** |
| **SLCO1B1** | SLCO1B1*5 | ***SLCO1B1*5*** | ***SLCO1B1*5*** | SLCO1B1*5 | SLCO1B1*5 | ***SLCO1B1*5*** | SLCO1B1*5 | SLCO1B1*5 |
| **TPMT** | TPMT *2,*3B,*3C,*4 | ***TPMT *2,*3B,*3C,*4*** | ***TPMT *2,*3B,*3C,*4*** | TPMT *2,*3B,*3C,*4 | TPMT *2,*3B,*3C,*4 | ***TPMT *2,*3B,*3C,*4*** | TPMT *2,*3B,*3C,*4 | TPMT *2,*3B,*3C,*4 |
| **UGT1A1** | UGT1A1*80 | UGT1A1*80 | UGT1A1*80 | UGT1A1*80 | UGT1A1*80 | ***UGT1A1*80*** | UGT1A1*80 | ***UGT1A1*80*** |
| **VKORC1** | VKORC1 *2 -c.1639 G>A,*2 6484G>T,*3 | ***VKORC1*** *2 -c.1639 G>A,****2 6484G>T,*3*** | ***VKORC1 *2 -c.1639 G>A***,*2 6484G>T,*3 | VKORC1 *2 -c.1639 G>A,*2 6484G>T,*3 | ***VKORC1 *2 -c.1639 G>A***,*2 6484G>T,*3 | ***VKORC1 *2 -c.1639 G>A,82 6484G>T,*3*** | VKORC1 *2 -c.1639 G>A,*2 6484G>T,*3 | VKORC1 *2 -c.1639 G>A,*2 6484G>T,*3 |

**
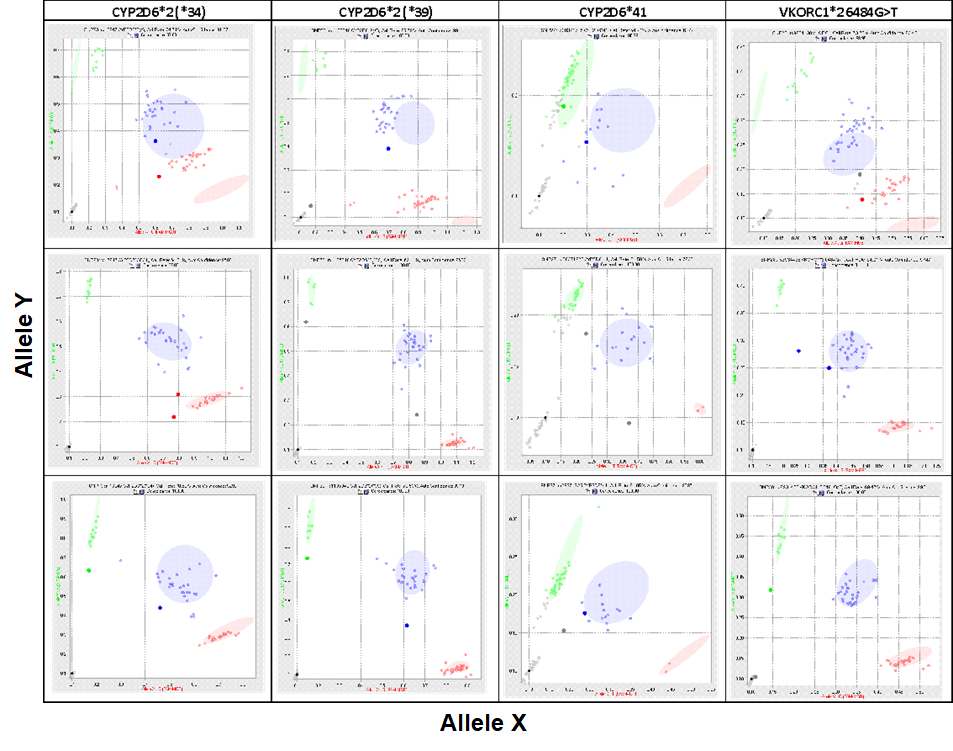
**

**Supplemental Figure 1. All discrepant calls for sample 18. The larger dots in each scatter plot indicate calls for sample 18. The first column indicates a run date and the initials of the tech performing the run. Sample 18 should be in the heterozygous (blue) clusters for CYP2D6*2 (*34), CYP2D6*2 (*39), CYP2D6*41 and VKORC1*2 6484G>T.**


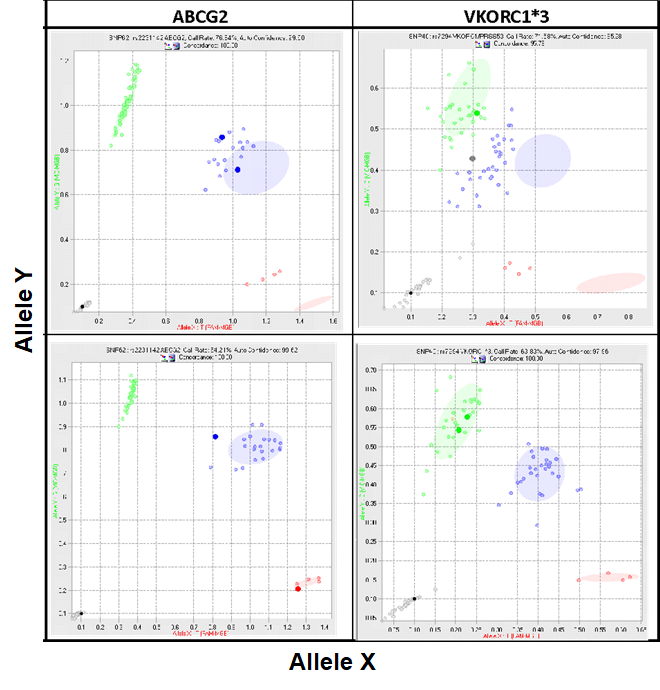


**Supplemental Figure 2. All discrepant calls for sample 23. The larger dots in each scatter plot indicate calls for sample 23. The first column indicates a run date and the initials of the tech performing the run. Sample should be in the heterozygous (blue) cluster for ABCG2 and the homozygous green cluster for VKORC1*3.**


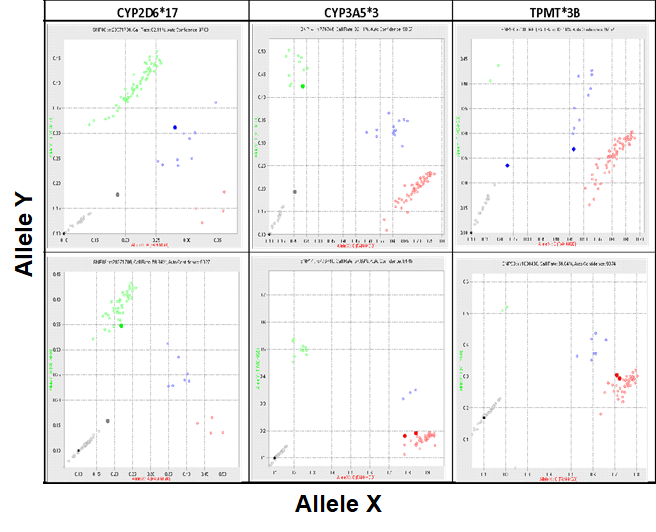


**Supplemental Figure 3. All discrepant calls for sample 37. The larger dots in each scatter plot indicate calls for sample 37. The first column indicates a run date and the initials of the tech performing the run. Sample should be in the heterozygous (blue) clusters for CYP2D6*17 and CYP3A5*3 and the homozygous red cluster for TPMT*3B.**


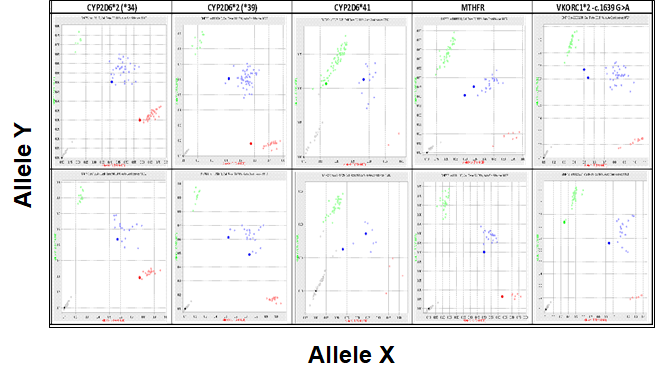


**Supplemental Figure 4. All discrepant calls for sample 40. The larger dots in each scatter plot indicate calls for sample 40. The first column indicates a run date and the initials of the tech performing the run. Sample should be in the heterozygous (blue) clusters for CYP2D6*2 (*34), CYP2D6*2 (*39), CYP2D6*41, MTHFR and VKORC1*2 –c.1639 G>A.**


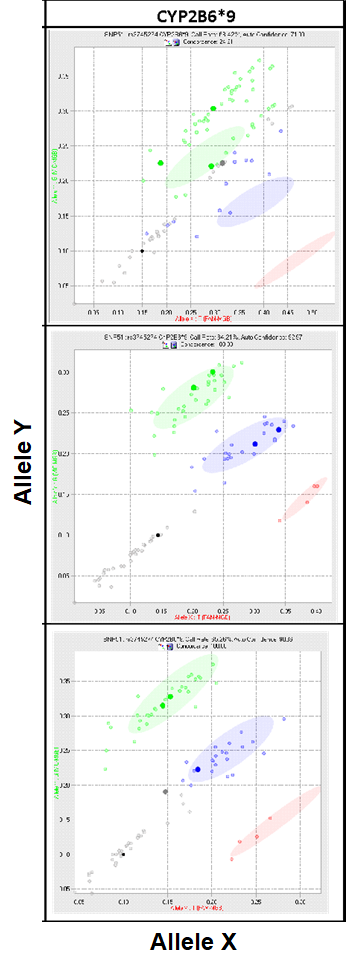


**Supplemental Figure 5. All discrepant calls for samples 154 and 163. The larger dots in each scatter plot indicate calls for 154 and 163. The first column indicates a run date and the initials of the tech performing the run. Sample 154 should be in the homozygous green cluster, while sample 163 should be in the heterozygous blue cluster for CYP2B6*9.** The 3 false positive calls were the result of that particular validation run being thermal cycled on the BioMark HD rather than with the superior FC1 cycler Doubling the primer/probe concentration into the reactions for this assay, as well as thermal cycling on a Fluidigm FC1 cycler rather than the BioMark HD, led to improved distinct fluorescence clusters for each sample allele.

**Supplemental Table 7. Accuracy calls for the PGx panel on 96.96 IFC.**

| **Sample**  **Number** | **TP** | **TN** | **FP** | **FN** | **No Call** |
| --- | --- | --- | --- | --- | --- |
| 1 | 2 | 38 | 0 | 0 | 0 |
| 2 | 8 | 32 | 0 | 0 | 0 |
| 3 | 6 | 34 | 0 | 0 | 0 |
| 4 | 36 | 202 | 0 | 0 | 2 |
| 5 | 2 | 2 | 0 | 0 | 0 |
| 6 | 36 | 204 | 0 | 0 | 0 |
| 7 | 54 | 180 | 0 | 0 | 6 |
| 8 | 0 | 4 | 0 | 0 | 0 |
| 9 | 60 | 177 | 0 | 0 | 3 |
| 10 | 2 | 2 | 0 | 0 | 0 |
| 11 | 66 | 174 | 0 | 0 | 0 |
| 12 | 0 | 4 | 0 | 0 | 0 |
| 13 | 48 | 192 | 0 | 0 | 0 |
| 14 | 66 | 170 | 0 | 0 | 4 |
| 15 | 0 | 4 | 0 | 0 | 0 |
| 16 | 66 | 172 | 0 | 0 | 2 |
| 17 | 10 | 72 | 0 | 0 | 0 |
| 18 | 20 | 198 | 5 | 4 | 13 |
| 19 | 6 | 34 | 0 | 0 | 0 |
| 20 | 2 | 8 | 0 | 0 | 0 |
| 21 | 0 | 2 | 0 | 0 | 0 |
| 22 | 2 | 0 | 0 | 0 | 0 |
| 23 | 29 | 208 | 2 | 0 | 1 |
| 24 | 12 | 70 | 0 | 0 | 0 |
| 25 | 2 | 2 | 0 | 0 | 0 |
| 26 | 6 | 50 | 0 | 0 | 26 |
| 27 | 2 | 2 | 0 | 0 | 0 |
| 28 | 0 | 4 | 0 | 0 | 0 |
| 29 | 8 | 74 | 0 | 0 | 0 |
| 30 | 2 | 2 | 0 | 0 | 0 |
| 31 | 8 | 16 | 0 | 0 | 0 |
| 32 | 2 | 2 | 0 | 0 | 0 |
| 33 | 18 | 64 | 0 | 0 | 0 |
| 34 | 20 | 100 | 0 | 0 | 0 |
| 35 | 20 | 100 | 0 | 0 | 0 |
| 36 | 24 | 94 | 0 | 0 | 2 |
| 37 | 5 | 71 | 4 | 2 | 38 |
| 38 | 32 | 88 | 0 | 0 | 0 |
| 39 | 24 | 96 | 0 | 0 | 0 |
| 40 | 25 | 85 | 4 | 2 | 4 |
| 41 | 28 | 92 | 0 | 0 | 0 |
| 42 | 16 | 104 | 0 | 0 | 0 |
| 43 | 32 | 88 | 0 | 0 | 0 |
| 44 | 2 | 0 | 0 | 0 | 0 |
| 45 | 2 | 2 | 0 | 0 | 0 |
| 46 | 2 | 2 | 0 | 0 | 0 |
| 47 | 0 | 4 | 0 | 0 | 0 |
| 48 | 0 | 2 | 0 | 0 | 0 |
| 49 | 0 | 4 | 0 | 0 | 0 |
| 50 | 0 | 4 | 0 | 0 | 0 |
| 51 | 0 | 4 | 0 | 0 | 0 |
| 52 | 0 | 4 | 0 | 0 | 0 |
| 53 | 2 | 0 | 0 | 0 | 0 |
| 54 | 0 | 2 | 0 | 0 | 0 |
| 55 | 0 | 4 | 0 | 0 | 0 |
| 56 | 0 | 4 | 0 | 0 | 0 |
| 57 | 0 | 4 | 0 | 0 | 0 |
| 58 | 0 | 4 | 0 | 0 | 0 |
| 59 | 2 | 0 | 0 | 0 | 0 |
| 60 | 2 | 0 | 0 | 0 | 0 |
| 61 | 0 | 2 | 0 | 0 | 0 |
| 62 | 0 | 4 | 0 | 0 | 0 |
| 63 | 0 | 2 | 0 | 0 | 0 |
| 64 | 0 | 4 | 0 | 0 | 0 |
| 65 | 0 | 4 | 0 | 0 | 0 |
| 66 | 0 | 2 | 0 | 0 | 0 |
| 67 | 0 | 4 | 0 | 0 | 0 |
| 68 | 0 | 4 | 0 | 0 | 0 |
| 69 | 0 | 4 | 0 | 0 | 0 |
| 70 | 0 | 4 | 0 | 0 | 0 |
| 71 | 0 | 4 | 0 | 0 | 0 |
| 72 | 0 | 4 | 0 | 0 | 0 |
| 73 | 4 | 0 | 0 | 0 | 0 |
| 74 | 0 | 2 | 0 | 0 | 0 |
| 75 | 0 | 4 | 0 | 0 | 0 |
| 76 | 0 | 4 | 0 | 0 | 0 |
| 77 | 0 | 4 | 0 | 0 | 0 |
| 78 | 0 | 2 | 0 | 0 | 0 |
| 79 | 2 | 0 | 0 | 0 | 0 |
| 80 | 4 | 2 | 0 | 0 | 0 |
| 81 | 0 | 4 | 0 | 0 | 0 |
| 82 | 0 | 2 | 0 | 0 | 0 |
| 83 | 0 | 4 | 0 | 0 | 0 |
| 84 | 0 | 2 | 0 | 0 | 0 |
| 85 | 0 | 4 | 0 | 0 | 0 |
| 86 | 0 | 2 | 0 | 0 | 0 |
| 87 | 2 | 0 | 0 | 0 | 0 |
| 88 | 42 | 190 | 0 | 0 | 8 |
| 89 | 2 | 2 | 0 | 0 | 0 |
| 90 | 2 | 2 | 0 | 0 | 0 |
| 91 | 2 | 2 | 0 | 0 | 0 |
| 92 | 2 | 2 | 0 | 0 | 0 |
| 93 | 0 | 4 | 0 | 0 | 0 |
| 94 | 2 | 0 | 0 | 0 | 0 |
| 95 | 2 | 2 | 0 | 0 | 0 |
| 96 | 2 | 2 | 0 | 0 | 0 |
| 97 | 0 | 4 | 0 | 0 | 0 |
| 98 | 2 | 2 | 0 | 0 | 0 |
| 99 | 2 | 38 | 0 | 0 | 0 |
| 100 | 16 | 8 | 0 | 0 | 0 |
| 101 | 2 | 0 | 0 | 0 | 0 |
| 102 | 2 | 0 | 0 | 0 | 0 |
| 103 | 0 | 4 | 0 | 0 | 0 |
| 104 | 0 | 4 | 0 | 0 | 0 |
| 105 | 0 | 4 | 0 | 0 | 0 |
| 106 | 2 | 2 | 0 | 0 | 0 |
| 107 | 8 | 8 | 0 | 0 | 0 |
| 108 | 0 | 4 | 0 | 0 | 0 |
| 109 | 0 | 2 | 0 | 0 | 0 |
| 110 | 2 | 2 | 0 | 0 | 0 |
| 111 | 2 | 2 | 0 | 0 | 0 |
| 112 | 0 | 4 | 0 | 0 | 0 |
| 113 | 0 | 2 | 0 | 0 | 0 |
| 114 | 0 | 4 | 0 | 0 | 0 |
| 115 | 2 | 4 | 0 | 0 | 0 |
| 116 | 0 | 4 | 0 | 0 | 0 |
| 117 | 0 | 4 | 0 | 0 | 0 |
| 118 | 0 | 4 | 0 | 0 | 0 |
| 119 | 12 | 70 | 0 | 0 | 0 |
| 120 | 2 | 2 | 0 | 0 | 0 |
| 121 | 12 | 70 | 0 | 0 | 0 |
| 122 | 12 | 69 | 0 | 0 | 1 |
| 123 | 10 | 72 | 0 | 0 | 0 |
| 124 | 20 | 62 | 0 | 0 | 0 |
| 125 | 2 | 38 | 0 | 0 | 0 |
| 126 | 10 | 32 | 0 | 0 | 0 |
| 127 | 6 | 36 | 0 | 0 | 0 |
| 128 | 2 | 2 | 0 | 0 | 0 |
| 129 | 2 | 2 | 0 | 0 | 0 |
| 130 | 2 | 2 | 0 | 0 | 0 |
| 131 | 2 | 2 | 0 | 0 | 0 |
| 132 | 28 | 131 | 0 | 0 | 5 |
| 133 | 56 | 263 | 0 | 0 | 1 |
| 134 | 20 | 76 | 0 | 0 | 0 |
| 135 | 26 | 70 | 0 | 0 | 0 |
| 136 | 40 | 279 | 0 | 0 | 1 |
| 137 | 48 | 259 | 0 | 0 | 13 |
| 138 | 80 | 312 | 0 | 0 | 8 |
| 139 | 64 | 255 | 0 | 0 | 1 |
| 140 | 64 | 256 | 0 | 0 | 0 |
| 141 | 48 | 268 | 0 | 0 | 4 |
| 142 | 64 | 255 | 0 | 0 | 1 |
| 143 | 12 | 32 | 0 | 0 | 0 |
| 144 | 12 | 32 | 0 | 0 | 0 |
| 145 | 30 | 102 | 0 | 0 | 0 |
| 146 | 30 | 102 | 0 | 0 | 0 |
| 147 | 42 | 89 | 0 | 0 | 1 |
| 148 | 30 | 102 | 0 | 0 | 0 |
| 149 | 18 | 113 | 0 | 0 | 1 |
| 150 | 42 | 106 | 0 | 0 | 2 |
| 151 | 48 | 83 | 0 | 0 | 1 |
| 152 | 42 | 117 | 0 | 0 | 3 |
| 153 | 24 | 105 | 0 | 0 | 3 |
| 154 | 48 | 101 | 1 | 0 | 0 |
| 155 | 12 | 32 | 0 | 0 | 0 |
| 156 | 12 | 32 | 0 | 0 | 0 |
| 157 | 12 | 32 | 0 | 0 | 0 |
| 158 | 36 | 53 | 0 | 0 | 3 |
| 159 | 48 | 84 | 0 | 0 | 6 |
| 160 | 36 | 96 | 0 | 0 | 0 |
| 161 | 28 | 100 | 0 | 0 | 4 |
| 162 | 4 | 39 | 0 | 0 | 1 |
| 163 | 32 | 104 | 2 | 0 | 6 |
| 164 | 22 | 74 | 0 | 0 | 0 |
| 165 | 32 | 124 | 0 | 0 | 4 |
| 166 | 66 | 173 | 0 | 0 | 1 |
| 167 | 72 | 247 | 0 | 0 | 1 |
| 168 | 56 | 264 | 0 | 0 | 0 |
| **Total** | **2405** | **9176** | **18** | **8** | **181** |

**Supplemental Table 8. List of gene-drug recommendations on the clinically implemented assay.**

| **Drug name** | **Drug Class** | **Therapeutic Class** | **Specialty** |
| --- | --- | --- | --- |
| celecoxib | COX2 inhibitor | Antiinflammatories | Analgesia |
| codeine | Opioid | Opioids | Analgesia |
| flurbiprofen | NSAID | Antiinflammatories | Analgesia |
| ibuprofen | NSAID | Antiinflammatories | Analgesia |
| lornoxicam | NSAID | Antiinflammatories | Analgesia |
| meloxicam | NSAID | Antiinflammatories | Analgesia |
| piroxicam | NSAID | Antiinflammatories | Analgesia |
| tenoxicam | NSAID | Antiinflammatories | Analgesia |
| clopidogrel | P2Y12 Inhibitor | Antithrombotics | Cardiovascular |
| simvastatin | Statin | Lipid-lowering medications | Cardiovascular |
| warfarin | Vitamin K antagonist | Antithrombotics | Cardiovascular |
| dexlansoprazole | Proton pump inhibitor | Acid-lowering medications | Gastroenterology |
| lansoprazole | Proton pump inhibitor | Acid-lowering medications | Gastroenterology |
| omeprazole | Proton pump inhibitor | Acid-lowering medications | Gastroenterology |
| ondansetron | 5HT3 antagonists | Antiemetics | Gastroenterology |
| pantoprazole | Proton pump inhibitor | Acid-lowering medications | Gastroenterology |
| tropisetron | 5HT3 antagonists | Antiemetics | Gastroenterology |
| azathioprine | Antimetabolites | Immunosuppressants | Immunology |
| tacrolimus | Calcineurin inhibitors | Immunosuppressants | Immunology |
| efavirenz | Non-nucleoside reverse transcriptase inhibitors | Antivirals | Infectious Disease |
| voriconazole | Azole antifungals | Antifungals | Infectious Disease |
| atazanavir | Protease inhibitors | Antivirals | Infectious disease |
| amitriptyline | Tricyclic antidepressant | Antidepressants | Neurology/  Psychiatry |
| atomoxetine | Non-stimulants | ADHD medications | Neurology/  Psychiatry |
| citalopram | Selective serotonin reuptake inhibitors | Antidepressants | Neurology/  Psychiatry |
| clomipramine | Tricyclic antidepressant | Antidepressants | Neurology/  Psychiatry |
| desipramine | Tricyclic antidepressant | Antidepressants | Neurology/  Psychiatry |
| doxepin | Tricyclic antidepressant | Antidepressants | Neurology/  Psychiatry |
| escitalopram | Selective serotonin reuptake inhibitors | Antidepressants | Neurology/  Psychiatry |
| fluvoxamine | Selective serotonin reuptake inhibitors | Antidepressants | Neurology/  Psychiatry |
| fosphenytoin | Hydantoin antiepileptics | Antiepileptics | Neurology/  Psychiatry |
| imipramine | Tricyclic antidepressant | Antidepressants | Neurology/  Psychiatry |
| nortriptyline | Tricyclic antidepressant | Antidepressants | Neurology/  Psychiatry |
| paroxetine | Selective serotonin reuptake inhibitors | Antidepressants | Neurology/  Psychiatry |
| phenytoin | Hydantoin antiepileptics | Antiepileptics | Neurology/  Psychiatry |
| trimipramine | Tricyclic antidepressant | Antidepressants | Neurology/  Psychiatry |
| sertraline | Selective serotonin reuptake inhibitors | Antidepressants | Neurology/  Psychiatry |
| capecitabine | Pyrimidine analogues | Antimetabolites | Oncology |
| fluorouracil | Pyrimidine analogues | Antimetabolites | Oncology |
| mercaptopurine | Purine analogues | Antimetabolites | Oncology |
| tamoxifen | Anti-estrogens | Hormone antagonists | Oncology |
| thioguanine | Purine analogues | Antimetabolites | Oncology |
